# Monitoring hyperproperties

**DOI:** 10.1007/s10703-019-00334-z

**Published:** 2019-06-25

**Authors:** Bernd Finkbeiner, Christopher Hahn, Marvin Stenger, Leander Tentrup

**Affiliations:** Reactive Systems Group, Saarland University, Saarbrücken, Germany

**Keywords:** Hyperproperties, Runtime verification, Monitoring, Information-flow

## Abstract

Hyperproperties, such as non-interference and observational determinism, relate multiple system executions to each other. They are not expressible in standard temporal logics, like LTL, CTL, and CTL*, and thus cannot be monitored with standard runtime verification techniques. $\text {HyperLTL}$ extends linear-time temporal logic (LTL) with explicit quantification over traces in order to express hyperproperties. We investigate the runtime verification problem of $\text {HyperLTL}$ formulas for three different input models: (1) The *parallel* model, where a *fixed* number of system executions is processed in parallel. (2) The *unbounded sequential* model, where system executions are processed sequentially, one execution at a time. In this model, the number of incoming executions is a-priori unbounded and may in fact grow forever. (3) The *bounded sequential model* where the traces are processed sequentially and the number of incoming executions is *bounded*. We show that the existence of a bound in the parallel and bounded sequential models leads to a different notion of monitorability than in the unbounded sequential model. We show that deciding the monitoriability problem for alternation-free HyperLTL is $\textsc {PSpace}$-complete while the problem is undecidable in general. For every input model, we provide monitoring algorithms along with run-time and storage optimizations. By recognizing properties of specifications such as reflexivity, symmetry, and transitivity, we reduce the number of comparisons between traces. For the sequential models, we present a technique that minimizes the number of traces that need to be stored. We evaluate our optimizations, showing that this leads to a more scalable monitoring and, in particular, a significantly lower memory consumption.

## Introduction

*Hyperproperties* [13] generalize trace properties in that they not only check the correctness of individual traces, but can also relate multiple computation traces to each other. This is needed, for example, to express information flow security policies like the requirement that the system behavior appears to be deterministic, i.e., independent of certain secrets, to an external observer. Monitoring hyperproperties is difficult, because it is no longer possible to analyze traces in isolation: a violation of a hyperproperty in general involves a set of traces, not just a single execution. We present monitoring algorithms for hyperproperties given in the temporal logic HyperLTL [12], which extends linear-time temporal logic (LTL) with trace variables and trace quantifiers in order to refer to multiple traces at a time. For example, the HyperLTL formula $\forall \pi . \exists \pi '.~ \Box dummyInput_{\pi '} \wedge ~ lowOut_\pi = lowOut_{\pi '}$ expresses *noninference* [28] by stating that for all traces $\pi$, there exists a trace $\pi '$, such that the observable outputs are the same on both traces even when the high security input of $\pi '$ being replaced by a dummy input. For example, in a messaging app, we might replace the address book, which we want to keep secret, with an *empty* address book.

A first, and absolutely fundamental, question to be answered is in what form the input, which now consists of more than one execution trace, should be presented to the monitor. Should the traces be presented all at once or one at a time? Is the number of traces known in advance? Obviously, the choice of the input representation has significant impact both on the principal monitoriability of a hyperproperty and on the actual monitoring algorithm. We study three basic input models for monitoring hyperproperties. (1) The *parallel* model, where a *fixed* number of system executions is processed in parallel. (2) The *unbounded sequential* model, where system executions are processed sequentially, one execution at a time. In this model, the number of incoming executions is a-priori unbounded and may in fact grow forever. (3) The *bounded sequential model* where the traces are processed sequentially and the number of incoming executions is *bounded*.

**Fig. 1:**
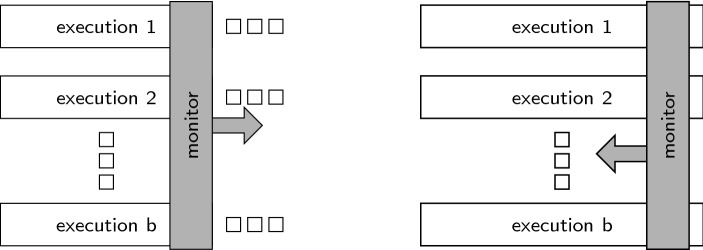
Monitor approaches for the parallel model: online in a forward fashion (left) and offline in a backwards fashion (right)

*Parallel model.* The assumption that the number of incoming traces is fixed before the actual monitoring process starts is a straight-forward extension of the classic runtime verification problem for LTL, where only a single trace is observed. We distinguish *online monitoring*, where the traces become available one position at a time from left to right, from *offline monitoring* where the positions of the traces can be accessed in any order. Figure 1 illustrates the two types of algorithms. The parallel model is known from techniques like secure-multi-execution [15], where several system executions are generated by providing different high-security inputs. We present an online and an offline monitoring algorithm for hyperproperties expressed in $\text {HyperLTL}$. The online algorithm is based on standard techniques for building monitoring automata from LTL formulas. Such a monitor automaton is then instantiated for multiple traces as specified by the HyperLTL formula. The offline algorithm is based on constructing an alternating automaton and then proceeding through the automaton in a bottom-up fashion, similar to the classic construction for LTL [23].

**Fig. 2:**
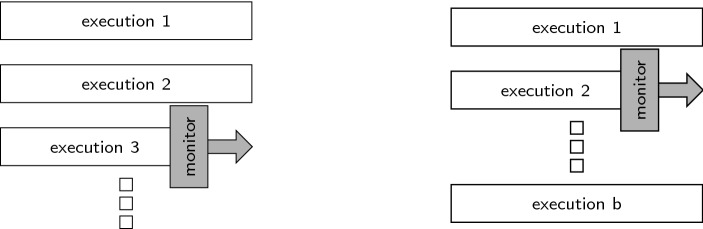
Monitor approaches for the sequential models: an unbounded number of traces (left) and bounded number of traces (right) are processed sequentially

*Sequential model.* The sequential models are useful when multiple sessions of a system under observation have to be monitored one after the other in an online fashion. The disadvantage of the unbounded sequential model is that many interesting hyperproperties, in particular most hyperproperties with quantifier alternations, are not monitorable in this model. It is therefore often useful to define a stop condition in the form of a bound on the number of traces that need to be handled during the monitoring process. Figure 2 sketches the monitoring algorithm for the unbounded and bounded cases.

A naive monitoring approach for the sequential models would be to simply store all traces seen so far. However, this would create two problems: a memory problem, because the needed memory grows with the number of traces observed by the monitor, and a runtime problem, because one needs to relate every newly observed trace against the growing set of stored traces.

There are hyperproperties where this effect cannot be avoided. An example is the hyperproperty with two atomic propositions *p* and *q*, where any pair of traces that agree on their *p* labeling must also agree on their *q* labeling. Clearly, for every *p* labeling seen so far, we must also store the corresponding *q* labeling. In practice, however, it is often possible to greatly simplify the monitoring. Consider, for example, the hyperproperty that states that all traces have the same *q* labeling (independently of the *p* labeling). In HyperLTL, this property is specified as the formula $\forall \pi .\forall \pi '.\Box (q_\pi {\leftrightarrow } q_{\pi '})$. The naive approach would store all traces seen so far, and thus requires, in the worst case, $O(t \cdot n)$ memory after *n* traces of length *t*. A new trace would be compared against every stored trace twice, once as $\pi$ and once as $\pi '$, resulting in an $O(t \cdot n)$ running time for each new trace. Obviously, however, in this example it is sufficient to store the first trace, and compare all further incoming traces against this reference. The required memory is thus, in fact, constant in the number of traces. A further observation is that the specification is symmetric in $\pi$ and $\pi '$. Hence, a single comparison suffices.

In this article, we present a monitoring approach for hyperproperties in the unbounded model that reduces the set of traces that new traces must be compared against to a minimal subset. Our approach comes with a strong correctness guarantee: our monitor produces the same verdict as a naive monitor that would store all traces and, additionally, we keep a sufficient set of traces to always provide an actually observed witness for the monitoring verdict. Our monitoring algorithm thus delivers a result that is equally informative as the naive solution. We introduce two analysis techniques: *The trace analysis* reduces the stored set of traces to a minimum, thus minimizing the required memory. *The specification analysis*, which is applicable in the parallel model as well, identifies symmetry, transitivity, and reflexivity in the specification, in order to reduce the algorithmic workload that needs to be carried out on the stored traces.

*Trace analysis* As an example for a system where information flow control is of outstanding importance for the intended operation, we consider a conference management system. There are a number of confidentiality properties that such a system should satisfy, like *“The final decision of the program committee remains secret until the notification”*. We want to focus on important hyperproperties of interest beyond confidentiality, like the property that no paper submission is lost or delayed. Informally, one formulation of this property is *“A paper submission is immediately visible for every program committee member”*. More formally, this property relates pairs of traces, one belonging to an author and one belonging to a program committee member. We assume this separation is indicated by a proposition *pc* that is either disabled or enabled in the first component of those traces. Further propositions in our example are the proposition *s*, denoting that a paper has been submitted, and *v* denoting that the paper is visible.

Given a set of finite traces *T*, i.e., finite sequences over sets of atomic propositions, we can verify that the property holds by checking for every pair of traces $(t,t') \in T \times T$ with $pc \notin t[0]$ and $pc \in t'[0]$ that $s \in t[i]$ implies $v \in t'[i+1]$ for every $i \ge 0$. When *T* satisfies the property, $T \cup \{t^*\}$, where $t^*$ is a new trace, amounts to checking new pairs $(t^*,t)$ and $(t,t^*)$ for $t \in T$. This, however, leads to an increasing size of *T* and thereby to an increased number of checks: the monitoring problem inevitably becomes costlier over time. To circumvent this, we present a method that keeps the set of traces *minimal* with respect to the underlying property. When monitoring hyperproperties, traces may pose *requirements* on future traces. The core idea of our approach is to characterize traces that pose strictly stronger requirements on future traces than others. In this case, the traces with the weaker requirements can be safely discarded. As an example, consider the following set of traces 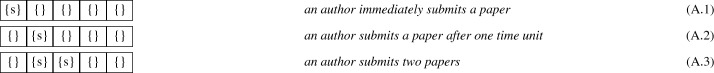
A satisfying PC trace would be $\{pc\}\{v\}\{v\}\{v\}\emptyset$ as there are author traces with paper submissions at time step 0, 1, and 2. For checking our property, one can safely discard trace A.2 as it poses no more requirements than trace A.3. We say that trace A.3 dominates trace A.2. We show that, given a property in the temporal logic $\text {HyperLTL}$, we can automatically reduce trace sets to be minimal with respect to this dominance. On relevant and more complex information flow properties, this reduces the memory consumption dramatically.

*Specification analysis* Our specification analysis technique allows us to reduce the number of monitor instantiations in order to detect violation or satisfaction of a given $\text {HyperLTL}$ formula. We use the decision procedure for the satisfiability problem of $\text {HyperLTL}$ [18] to check whether or not a universally quantified $\text {HyperLTL}$ formula is symmetric, transitive, or reflexive. If a hyperproperty is *symmetric*, then we can omit every symmetric monitor, thus, performing only half of the language membership tests. A canonical example for a symmetric $\text {HyperLTL}$ formula is $\forall \pi . \forall \pi '.\; (O_\pi = O_{\pi '}) {\mathcal {W}}(I_\pi \ne I_{\pi '}),$ a variant of observational determinism [27, 31, 38]. Symmetry is especially interesting, since many information flow policies have this property. If a hyperproperty is *transitive*, then we can omit every, except for one, monitor, since we can check every incoming trace against any reference trace. One example for a transitive $\text {HyperLTL}$ formula is equality $\forall \pi . \forall \pi ' .\Box (a_\pi \leftrightarrow a_{\pi '}).$ If a hyperproperty is *reflexive*, then we can omit the monitor where every trace variable i same trace. For example, both hyperproperties above are reflexive.

*Structure of this article* The remainder of this article is structured as follows. Section 2 introduces the syntax and semantics of $\text {HyperLTL}$ and the notion of monitorable $\text {HyperLTL}$ formula in all three input models. We furthermore present algorithms for checking whether a $\text {HyperLTL}$ formula is monitorable or not. In Sect. 3, we give a finite trace semantics for $\text {HyperLTL}$. For the parallel input model, we present an offline and an online monitoring algorithm for arbitrary $\text {HyperLTL}$ formulas. In Sect. 4, we present online algorithms for (universal) $\text {HyperLTL}$ formulas in the (unbounded) sequential model. We then tackle the aforementioned memory explosion by formally introducing the trace analysis and hyperproperty analysis sketched above. We report on our implementation RVHyper and experimental results in Sect. 5, before concluding in Sect. 6.

This is a revised and extended version of a paper that appeared at RV 2017 [20]. Our contributions and extensions compared to [20] are (1) the study of the above mentioned different runtime verification models for hyperproperties, (2) an online and offline, using backwards trace traversal, algorithm for the parallel model, and (3) an extended evaluation of an improved version of RVHyper.

*Related work* The temporal logic $\text {HyperLTL}$ was introduced to model check security properties of reactive systems [12, 22]. For one of its predecessors, $\text {SecLTL}$ [16], there has been a proposal for a white box monitoring approach [17] based on alternating automata. The problem of monitoring $\text {HyperLTL}$ has been considered before [1, 9]. Agrawal and Bonakdarpour [1] were the first to study the monitoring problem of HyperLTL for the sequential model. They focused on *k*-safety hyperproperties [13], where a hyperproperty $\varphi$ is *k*-safety, if for each set of traces violating $\varphi$ a bad set of prefixes of those traces can be given with at most *k* elements. In their paper Agrawal and Bonakdarpour show that HyperLTL formulas with at most *k* universal quantifiers can express a rich subset of *k*-safety hyperproperties. Following this observation they defined the notion of monitorability for HyperLTL based on the definitions for LTL monitorability by Pnueli and Zaks [30]. The concept of a monitorability formalizes the intuitive idea of being able to detect acceptance or violation of a property by only observing a system at runtime. So the monitorable HyperLTL formulas are those for which it is either possible to find a witness, a set of finite prefixes, of acceptance or violation, respectively. They gave a syntactic characterization of monitorable $\text {HyperLTL}$ formulas, which we extend in this work. The monitoring algorithm they propose is based on a progression logic expressing trace inter-dependencies and the composition of $\hbox {LTL}_3$ monitor automata in a Petri net whose special sink states represent verdicts for sub-formulas. Incoming traces get evaluated for each $\hbox {LTL}_3$ monitor in the Petri net and the progression logic keeps track of seen events for monitoring future traces. In subsequent work, a constraint based approach has been proposed [9]. By analyzing HyperLTL formulas syntactically, they identify propositions of interest and store corresponding constraints. Like with our monitoring algorithm, they do not have access to the implementation (black box), but in contrast to our work, they do not provide witnessing traces as a monitor verdict.

In [7], the authors study the complexity of monitoring hyperproperties. They show that the form and size of the input, as well as the formula have a significant impact on the feasibility of the monitoring process. They differentiate between several input forms and study their complexity: a set of linear traces, tree-shaped Kripke structures, and acyclic Kripke structures. For acyclic structures and alternation-free HyperLTL formulas, the problems complexity gets as low as NC. In [8], the authors discuss examples where static analysis can be combined with runtime verification techniques to monitor HyperLTL formulas beyond the alternation-free fragment. They discuss the challenges in monitoring formulas beyond this fragment and lay the foundations towards a general method.

The need to store traces during runtime is not unique to hyperproperties. There has been, for example, work on verifying parametric trace properties [10] which takes each event in the parametric trace and distributes it to its corresponding trace slices. This slicing is, unlike our trace analysis, independent of the specification. This technique motivated the work on Quantified Event Automata [4] that can evaluate quantifiers over parameters. They cannot, however, express general hyperproperties as they cannot relate different parametric traces.

For certain information flow policies, like non-interference and some extensions, dynamic enforcement mechanisms have been proposed. Techniques for the enforcement of information flow policies include tracking dependencies at the hardware level [34], language-based monitors [2, 3, 6, 32, 36], and abstraction-based dependency tracking [11, 24, 25]. Secure multi-execution [15] is a technique that can enforce non-interference by executing a program multiple times in different security levels. To enforce non-interference, the inputs are replaced by default values whenever a program tries to read from a higher security level.

## Runtime verification of $\text {HyperLTL}$

As hyperproperties relate multiple executions to each other, a monitor for hyperproperties has to consider sets of traces instead of solely processing a single execution in isolation. In this section, we elaborate on the runtime verification problem of $\text {HyperLTL}$. In the first subsection, we present $\text {HyperLTL}$, which is a temporal logic for expressing hyperproperties. In the second subsection, we define the notion of monitorable $\text {HyperLTL}$ specifications for three different input models: the unbounded input model, the bounded model, and the parallel model.

We start by giving necessary notation. Given a finite or infinite set *A*, we denote the powerset of a *A* by ${\mathcal {P}}(A)$ and define ${\mathcal {P}}^*(A)$ to be the set of all finite subsets of *A*. Note that ${\mathcal {P}}(A) = {\mathcal {P}}^*(A)$ if *A* is finite. For some $b > 0$ and infinite set *A*, we use the notation ${\mathcal {P}}^b(A)$ and ${\mathcal {P}}^{\le b}(A)$ to denote all subsets of *A* of cardinality *b* and $\le b$, respectively. Let $\text {AP}$ be a finite set of atomic propositions and let $\varSigma = {\mathcal {P}}(\text {AP})$ be the corresponding finite *alphabet*. A finite (infinite) trace is a finite (infinite) sequence over $\varSigma$. We denote the concatenation of a finite trace $u \in \varSigma ^*$ and a finite or infinite trace $v \in \varSigma ^* \cup \varSigma ^\omega$ by *uv* and write $u \preceq v$ if *u* is a prefix of *v*. Further, we lift the prefix operator to sets of traces, i.e., $U \preceq V$ if, and only if, $\forall u \in U .\exists v \in V .u \preceq v$ where $U \subseteq \varSigma ^*$ and $V \subseteq \varSigma ^* \cup \varSigma ^\omega$. Let *t* be a (infinite) trace and let $i,j \in {\mathbb {N}}$ be natural numbers with $j \ge i$. *t*[*i*] denotes the *i*-th element of *t*. Therefore, *t*[0] is the starting element of the trace. *t*[*i*, *j*] is the sequence $t[i] t[i+1] \ldots t[j-1] t[j]$. Lastly, $t[i,\infty ]$ denotes the infinite suffix of *t* starting at position *i*.

### HyperLTL

$\text {HyperLTL}$ [12] is a temporal logic for specifying hyperproperties. It extends $\text {LTL}$ [29] by quantification over trace variables $\pi$ and a method to link atomic propositions to specific traces. The set of trace variables is ${\mathcal {V}}$. Formulas in $\text {HyperLTL}$ are given by the grammar

$$\begin{aligned} \varphi&{}::=\forall \pi .\varphi \mid \exists \pi .\varphi \mid \psi , \text { and}\\ \psi&{}::=a_\pi \mid \lnot \psi \mid \psi \vee \psi \mid \Circle \psi \mid \psi {\mathcal {U}}\psi , \end{aligned}$$

where $a \in \text {AP}$ and $\pi \in {\mathcal {V}}$. We call a $\text {HyperLTL}$ formula existential or universal, if it contains solely existential or universal quantifiers, respectively. The alternation-free fragment of $\text {HyperLTL}$ is defined as the union of all existential and universal formulas. We consider only closed formulas, i.e., formulas where every occurring trace variable $\pi \in {\mathcal {V}}$ is bound by a quantifier. The semantics is given by the satisfaction relation $\vDash$ given in the following. Let $\varPi : {\mathcal {V}}\rightarrow \varSigma ^\omega$ be a function that maps trace variables to traces. To assign a variable $\pi \in {\mathcal {V}}$ to a trace $t \in \varSigma ^\omega$, we use the notation $\varPi [\pi \mapsto t]$. The satisfaction of a HyperLTL formula $\varphi$ over a trace assignment $\varPi$ and a set of traces *T*, denoted by $(T,\varPi ,i) \vDash \varphi$, is defined as

$$\begin{aligned} \begin{array}{ll} (T,\varPi ,i) \vDash a_\pi \qquad \qquad &{} \text {if } a \in \varPi (\pi )[i] \\ (T,\varPi ,i) \vDash \lnot \varphi &{} \text {if } (T,\varPi ,i) \nvDash \varphi \\ (T,\varPi ,i) \vDash \varphi \vee \psi &{} \text {if } (T,\varPi ,i) \vDash \varphi \text { or } (T,\varPi ,i) \vDash \psi \\ (T,\varPi ,i) \vDash \Circle \varphi &{} \text {if } (T,\varPi ,i+1) \vDash \varphi \\ (T,\varPi ,i) \vDash \varphi {\mathcal {U}}\psi &{} \text {if } \exists j \ge i .(T,\varPi ,j) \vDash \psi \wedge \forall i \le k < j .(T,\varPi ,k) \vDash \varphi \\ (T,\varPi ,i) \vDash \exists \pi .\varphi &{} \text {if there is some } t \in T \text { such that } (T,\varPi [\pi \mapsto t],i) \vDash \varphi \\ (T,\varPi ,i) \vDash \forall \pi .\varphi &{} \text {if for all } t \in T \text { it holds that } (T,\varPi [\pi \mapsto t],i) \vDash \varphi . \end{array} \end{aligned}$$

We write $T \vDash \varphi$ for $(T,\{\},0) \vDash \varphi$ where $\{\}$ denotes the empty assignment. The hyperproperty represented by a $\text {HyperLTL}$ formula $\varphi$, denoted by ${\mathcal {H}}(\varphi )$, is the set $\{ T \subseteq \varSigma ^\omega \mid T \vDash \varphi \}$. Depending on the quantifier prefix of $\varphi$, ${\mathcal {H}}(\varphi )$ has the following properties.

#### Lemma 1

Given some existential HyperLTL formula $\varphi$ and some $T \subseteq \varSigma ^\omega$. If $T \in {\mathcal {H}}(\varphi )$, then for all $T' \subseteq \varSigma ^\omega$ with $T \subseteq T'$ it holds that $T' \in {\mathcal {H}}(\varphi )$.

#### Proof

Let $\psi$ be the quantifier-free part of $\varphi$. Fix some $T'$ with $T' \supseteq T$. As $T \in {\mathcal {H}}(\varphi )$, $T \vDash \varphi$ and there is a trace assignment $\varPi$ such that $(T, \varPi , 0) \vDash \psi$ by the HyperLTL semantics. Hence, $(T', \varPi , 0) \vDash \psi$ and $T' \vDash \varphi$. $\square$

#### Corollary 1

Given some universal HyperLTL formula $\varphi$ with quantifier-free part $\psi$ over trace variables $\{\pi _1,\ldots ,\pi _n\}$ and some $T \subseteq \varSigma ^\omega$. If $T \notin {\mathcal {H}}(\varphi )$, then for all $T' \subseteq \varSigma ^\omega$ with $T \subseteq T'$ it holds that $T' \notin {\mathcal {H}}(\varphi )$.

#### Proof

By semantics of HyperLTL, $T \notin {\mathcal {H}}(\varphi )$ is equivalent to $T \in {\mathcal {H}}(\exists \pi _1 \cdots \exists \pi _n .\lnot \psi )$. Thus, the statement follows by Lemma 1. $\square$

Let $\varphi$ be a $\text {HyperLTL}$ formula with trace variables ${\mathcal {V}}= \{\pi _1,\ldots ,\pi _k\}$ over alphabet $\varSigma$. We define $\varSigma _{\mathcal {V}}$ to be the alphabet where $p_\pi$ is interpreted as an atomic proposition for every $p \in \text {AP}$ and $\pi \in {\mathcal {V}}$. We denote by $\vDash _\text {LTL}$ the $\text {LTL}$ satisfaction relation over $\varSigma _{\mathcal {V}}$. For LTL formulas $\varphi$ over alphabet $\varSigma$, we use ${\mathcal {L}}(\varphi )$ to denote the language of $\varphi$, i.e., the set $\{ t \in \varSigma _{\mathcal {V}}^\omega \mid t \vDash _\text {LTL}\varphi \}$. By slight abuse of notation, we use ${\mathcal {L}}$ for the set of traces satisfying the quantifier-free part of a HyperLTL formula under LTL semantics.

#### Lemma 2

Let $\psi$ be the quantifier-free part of some HyperLTL formula $\varphi$ over trace variables ${\mathcal {V}}$. There is a trace assignment $\varPi$ such that $(\emptyset , \varPi , 0) \vDash \psi$ if, and only if, $\psi$ is satisfiable under $\text {LTL}$ semantics over atomic propositions $\varSigma _{\mathcal {V}}$.

#### Proof

Assume that there is a trace assignment $\varPi$ over trace variables ${\mathcal {V}}$ such that $(\emptyset , \varPi , 0) \vDash \psi$. We define $w \in \varSigma _{\mathcal {V}}^\omega$ such that $p_\pi \in w[i]$ if, and only if, $p \in \varPi (\pi )[i]$ for all $i \ge 0$, $p \in \text {AP}$, and $\pi \in {\mathcal {V}}$. An induction over $\psi$ shows that $w \vDash _\text {LTL}\psi$.

Assume $\psi$ is satisfiable with respect to $\vDash _\text {LTL}$, i.e., there exists a $w \subseteq \varSigma ^\omega _{\mathcal {V}}$, such that $w \vDash _\text {LTL}\psi$. We define the $\pi$-projection, denoted by $\#_\pi (s)$, for a given $s \subseteq \varSigma _{\mathcal {V}}$ and $\pi \in {\mathcal {V}}$, as the set of all $p_\pi \in s$, formally, $\#_\pi (s) = \{ p \in \text {AP}\mid p_\pi \in s \}$. We construct an assignment $\varPi$ in the following manner: For any $\pi \in {\mathcal {V}}$, we map $\pi$ to the trace *t* obtained by projecting the corresponding $p_\pi \in \varSigma _{\mathcal {V}}$, i.e., $\forall i \ge 0 .t[i] = \#_\pi (w[i])$. $\square$

### Monitorability

The definition of monitorability for HyperLTL depends on the underlying runtime verification model, that is, the different ways how traces are given to the monitor. In this article, we distinguish between the sequential model [1, 9, 20], where traces are processed one-by-one, and the parallel model, where a fixed number of executions are observed.

In the (unbounded) sequential model, the size of the trace set is not known in advance. Traces are processed sequentially, i.e., given a finite set of traces *T* and a fresh trace *t*, the monitoring algorithm has to decide whether $T \cup \{t\}$ satisfies or violates a given $\text {HyperLTL}$ formula before processing the next trace $t'$. As a special case, we consider a variant of the sequential model where a maximal bound on the trace set is known. In the parallel model, we assume that the set of execution traces is of fixed size and, furthermore, that the traces are given at the same time. This corresponds, for example, to secure multi execution [15], where multiple instances of a system are run in parallel.

In the remainder of this section, we present notions for monitorability of hyperproperties for the respective input models. Those results extend earlier characterizations based on restricted syntactic fragments of $\text {HyperLTL}$ [1].

*Monitorability* For trace languages, monitorability is the property whether language containment can be decided by finite prefixes [30]. We fix some trace language $L \subseteq \varSigma ^\omega$. To characterize monitorability, one distinguishes two types of prefixes: a prefix is *good* if every infinite continuation results in a trace that is in the language *L*, analogously, a prefix is *bad* if no continuation is in the language. The set of good and bad prefixes is $\textit{good}(L) :=\{u \in \varSigma ^* \mid \forall v \in \varSigma ^\omega .uv \in L\}$ and $\textit{bad}(L) :=\{u \in \varSigma ^* \mid \forall v \in \varSigma ^\omega .uv \notin L\}$, respectively. A trace language *L* is *monitorable* if every prefix has a (finite) continuation that is either good or bad, formally, $\forall u \in \varSigma ^* .\exists v \in \varSigma ^* .uv \in \textit{good}(L) \vee uv \in \textit{bad}(L)$. The decision problem, i.e., given an $\text {LTL}$ formula $\varphi$, decide whether $\varphi$ is monitorable, is $\textsc {PSpace}$-complete [5].

A *hyperpropertyH* is a set of trace properties, i.e., $H \subseteq {\mathcal {P}}(\varSigma ^\omega )$. We fix some hyperproperty $H \subseteq {\mathcal {P}}(\varSigma ^\omega )$. Analogous to trace languages, we say that a finite set of prefix traces is *good* if every continuation, i.e., a (possibly infinite) set of infinite traces, is contained in *H*. The set of *good* and *bad prefix traces* is then formally defined as $\textit{good}(H) :=\{U \in {\mathcal {P}}^*(\varSigma ^*) \mid \forall V \in {\mathcal {P}}(\varSigma ^\omega ) .U \preceq V \Rightarrow V \in H\}$ and $\textit{bad}(H) :=\{U \in {\mathcal {P}}^*(\varSigma ^*) \mid \forall V \in {\mathcal {P}}(\varSigma ^\omega ) .U \preceq V \Rightarrow V \notin H\}$, respectively. The set of good and bad prefix traces inherit the properties given in Lemma 1 and Corollary 1.

#### Corollary 2

1. Given some existential HyperLTL formula $\varphi$ and some $T \subseteq \varSigma ^*$. If $T \in \textit{good}({\mathcal {H}}(\varphi ))$, then for all $T' \subseteq \varSigma ^*$ with $T \subseteq T'$ it holds that $T' \in \textit{good}({\mathcal {H}}(\varphi ))$.
2. Given some universal HyperLTL formula $\varphi$ and some $T \subseteq \varSigma ^*$. If $T \in \textit{bad}({\mathcal {H}}(\varphi ))$, then for all $T' \subseteq \varSigma ^*$ with $T \subseteq T'$ it holds that $T' \in \textit{bad}({\mathcal {H}}(\varphi ))$.

In this section, we will often use the translation between reasoning in HyperLTL (sets of traces) and LTL given in the proof of Lemma 2. In order to apply this translation for finite traces, we have to ensure that all traces have the same length. In the following, we state that this is the case for $\textit{bad}$ and $\textit{good}$.

#### Corollary 3

1. Given some existential HyperLTL formula $\varphi$ and some $T \subseteq \varSigma ^*$. If $T \in \textit{good}({\mathcal {H}}(\varphi ))$, then for all $T' \subseteq \varSigma ^*$ with $T \preceq T'$, ${|T|} = {|T'|}$, and ${|t|} = {|t'|}$ for all $t,t' \in T'$ it holds that $T' \in \textit{good}({\mathcal {H}}(\varphi ))$.
2. Given some universal HyperLTL formula $\varphi$ and some $T \subseteq \varSigma ^*$. If $T \in \textit{bad}({\mathcal {H}}(\varphi ))$, then for all $T' \subseteq \varSigma ^*$ with $T \preceq T'$, ${|T|} = {|T'|}$, and ${|t|} = {|t'|}$ for all $t,t' \in T'$ it holds that $T' \in \textit{bad}({\mathcal {H}}(\varphi ))$.

*Unbounded sequential model* A hyperproperty *H* is *monitorable* in the unbounded input model [1] if every finite prefix set has a good or bad continuation, formally,

$$\begin{aligned} \forall U \in {\mathcal {P}}^*(\varSigma ^*) .\exists V \in {\mathcal {P}}^*(\varSigma ^*) .U \preceq V \wedge \big ( V \in \textit{good}(H) \vee V \in \textit{bad}(H) \big ). \end{aligned}$$

#### Example 1

As an example, consider trace equality expressed by the HyperLTL formula $\varphi _eq = \forall \pi . \forall \pi ' .\Box (a_\pi \leftrightarrow a_{\pi '})$. To prove monitorability, we pick some arbitrary prefix set $U \in {\mathcal {P}}^*(\varSigma ^*)$. Based on *U* we construct a continuation *V* (with $U \preceq V$) such that $V \in \textit{bad}({\mathcal {H}}(\varphi _eq ))$. In case $U = \emptyset$ or $U = \{\epsilon \}$, we choose $V = \{ \{a\}\emptyset ^\omega , \emptyset ^\omega \}$, otherwise, pick some $u \in U$ where $u \ne \epsilon$. We choose $V = U \cup \{ u' \}$ where $u'$ is a copy of *u* except for the first position, that is, $u'_i = u_i$ for $0< i < {|u|}$ and $u'_0 = \emptyset$ if $a \in u_0$ and $u'_0 = \{a\}$ otherwise. By construction, it holds that $U \preceq V$ and, further, $V \in \textit{bad}(\varphi _eq )$ as there are two traces in *V* that differ on the valuation of *a* in the first position.

With this definition, hardly any alternating $\text {HyperLTL}$ formula is monitorable as their satisfaction cannot be characterized by a finite trace set, even for safety properties. Consider, for example, the formula $\varphi _{\textit{alt}} = \forall \pi .\exists \pi ' .\Box (a_\pi \rightarrow b_{\pi '})$. Assume a finite set of traces *T*. One can construct a new trace *t* where $a \in t[i]$ and $b \notin t[i]$ for some position *i* such that $b \notin t'[i]$ for all traces $t' \in T$. Thus, the continuation of this trace set (which does not add traces) violates $\varphi _{\textit{alt}}$. Likewise, a sufficiently long trace containing only *b*’s leads to a satisfied continuation. Thus, $\varphi _{\textit{alt}}$ has neither good nor bad prefix traces.

In the following, we develop a method to decide whether an alternation-free $\text {HyperLTL}$ formula is monitorable. This fragment includes a wide range of hypersafety [13] properties, such as non-interference. We start by showing that a universal HyperLTL formula has no good prefix traces except for the special case where the quantifier-free part is equivalent to $\textit{true}$.

#### Lemma 3

Let $\psi$ be the quantifier-free part of some $\forall ^n\text {HyperLTL}$ formula $\varphi$ over trace variables ${\mathcal {V}}$ for some $n > 0$. It holds that $\textit{good}({\mathcal {H}}(\varphi )) = \emptyset$ unless ${\mathcal {L}}(\psi ) = \varSigma _{\mathcal {V}}^\omega$.

#### Proof

If ${\mathcal {L}}(\psi ) = \varSigma _{\mathcal {V}}^\omega$ then ${\mathcal {H}}(\varphi ) = {\mathcal {P}}(\varSigma ^\omega )$ and $\textit{good}({\mathcal {H}}(\varphi )) = {\mathcal {P}}^*(\varSigma ^*)$. Assume for contradiction that ${\mathcal {L}}(\psi ) \ne \varSigma _{\mathcal {V}}^\omega$ and $\textit{good}({\mathcal {H}}(\varphi )) \ne \emptyset$, i.e., there is a finite set $U \subseteq \varSigma ^*$ that is a good prefix set of ${\mathcal {H}}(\varphi )$. Since ${\mathcal {L}}(\psi ) \ne \varSigma _{\mathcal {V}}^\omega$, there is at least one infinite trace $\sigma \in \varSigma _{\mathcal {V}}^\omega$ with $\sigma \nvDash _\text {LTL}\psi$. We translate this trace to a set of infinite traces *W* where $W \nvDash \varphi$ using the same construction as in Lemma 2. Let $V \in {\mathcal {P}}(\varSigma ^\omega )$ be an arbitrary continuation of *U*, i.e., $U \preceq V$. It holds that $U \preceq (V \cup W)$ and $V \cup W \notin {\mathcal {H}}(\varphi )$ by Corollary 1, hence, contradicting $U \in \textit{good}({\mathcal {H}}(\varphi ))$. $\square$

The following theorem characterizes the monitorability for universal HyperLTL formulas.

#### Theorem 1

Let $\psi$ be the quantifier-free part of some universal $\text {HyperLTL}$ formula $\varphi$ over trace variables ${\mathcal {V}}$. $\varphi$ is monitorable if, and only if, ${\mathcal {L}}(\psi ) = \varSigma _{\mathcal {V}}^\omega$ or $\textit{bad}({\mathcal {L}}(\psi )) \ne \emptyset$.

#### Proof

Assume that ${\mathcal {L}}(\psi ) = \varSigma _{\mathcal {V}}^\omega$. It holds that ${\mathcal {H}}(\varphi ) = {\mathcal {P}}(\varSigma ^\omega )$, thus, $\textit{good}({\mathcal {H}}(\varphi )) = {\mathcal {P}}^*(\varSigma ^*)$ immediately satisfying the monitorability definition by choosing $V=U$ for every $U \in {\mathcal {P}}^*(\varSigma ^*)$.

Assume $\textit{bad}({\mathcal {L}}(\psi )) \ne \emptyset$, thus there is some $v \in \varSigma _{\mathcal {V}}^*$ such that $v \in \textit{bad}({\mathcal {L}}(\psi ))$. Fix some arbitrary prefix $U \in {\mathcal {P}}^*(\varSigma ^*)$. Let $V \in {\mathcal {P}}^*(\varSigma ^*)$ be a prefix trace set corresponding to *v* using the same construction as used in the proof of Lemma 2. As *v* is a bad prefix, all continuations $w \in \varSigma _{\mathcal {V}}^\omega$ with $v \preceq w$ are not in ${\mathcal {L}}(\psi )$. Thus, by Lemma 2, the corresponding trace set $W \in {\mathcal {P}}^*(\varSigma ^\omega )$ is not in ${\mathcal {H}}(\varphi )$. Using Corollary 1 we conclude that for every $W' \in {\mathcal {P}}(\varSigma ^\omega )$ with $W \subseteq W'$ it holds that $W' \notin {\mathcal {H}}(\varphi )$, hence, *V* is a bad prefix (as $W'$ represents all possible continuations of *V* by construction) and thereby $V \cup U \in \textit{bad}({\mathcal {H}}(\varphi ))$ using the monotonicity of $\textit{bad}$ (Corollary 2).

Assume $\varphi$ is monitorable, thus, $\forall U \in {\mathcal {P}}^*(\varSigma ^*) .\exists V \in {\mathcal {P}}^*(\varSigma ^*) .U \preceq V \wedge \big ( V \in \textit{good}({\mathcal {H}}(\varphi )) \vee V \in \textit{bad}({\mathcal {H}}(\varphi )) \big )$. As the set of good prefixes $\textit{good}({\mathcal {L}}(\varphi ))$ is empty if ${\mathcal {L}}(\psi ) \ne \varSigma _{\mathcal {V}}^\omega$ by Lemma 3 we can simplify the formula to $\forall U \in {\mathcal {P}}^*(\varSigma ^*) .\exists V \in {\mathcal {P}}^*(\varSigma ^*) .U \preceq V \wedge V \in \textit{bad}({\mathcal {H}}(\varphi ))$ by assuming that ${\mathcal {L}}(\psi ) \ne \varSigma _{\mathcal {V}}^\omega$.

It remains to show that $\textit{bad}({\mathcal {L}}(\psi )) \ne \emptyset$. Picking some arbitrary *U*, e.g., $U = \emptyset$, we get some $V \in {\mathcal {P}}^*(\varSigma ^*)$ such that $U \preceq V$ and $V \in \textit{bad}({\mathcal {H}}(\varphi ))$. Let $V'$ be a bad prefix where all traces have the same length (Corollary 3). Thus, for all $W \in {\mathcal {P}}(\varSigma ^\omega )$ with $V' \preceq W$, it holds that $W \notin {\mathcal {H}}(\varphi )$. This holds especially if we restrict ${|W|} = {|V'|}$, which gives us the witnessing continuation $w \in \varSigma _{\mathcal {V}}^\omega$ (corresponding to *W*) to show that $v \in \varSigma _{\mathcal {V}}^*$ (corresponding to $V'$) is a bad prefix of ${\mathcal {L}}(\psi )$. It holds that $v \preceq w$ by construction, and $w \notin {\mathcal {L}}(\psi )$ due to Lemma 2. $\square$

#### Corollary 4

Let $\psi$ be the quantifier-free part of some existential $\text {HyperLTL}$ formula $\varphi$ over trace variables ${\mathcal {V}}$. $\varphi$ is monitorable if, and only if, ${\mathcal {L}}(\psi ) = \emptyset$ or $\textit{good}({\mathcal {L}}(\psi )) \ne \emptyset$.

#### Theorem 2

Given an alternation-free $\text {HyperLTL}$ formula $\varphi$. Deciding whether $\varphi$ is monitorable in the unbounded sequential model is $\textsc {PSpace}$-complete.

#### Proof

We consider the case that $\varphi$ is universal, the case for existentially quantified formulas is dual. We apply the characterization from Theorem 1. First, we have to check validity of $\psi$ which can be done in polynomial space [33]. Next, we have to determine whether $\textit{bad}({\mathcal {L}}(\psi ))$ is empty, which can be done by a $\textsc {PSpace}$ algorithm given by Bauer [5]. Hardness follows as the problem is already $\textsc {PSpace}$-hard for $\text {LTL}$. $\square$

In general, however, deciding monitorability for HyperLTL is undecidable. We show this by a reduction from the satisfiability problem [18].

#### Theorem 3

Deciding whether a $\text {HyperLTL}$ formula $\varphi$ is monitorable in the unbounded sequential model is undecidable.

#### Proof

We use a reduction from the satisfiability problem of HyperLTL [18]. In detail, given some HyperLTL formula $\varphi$, we construct a formula $\varphi '$ that is monitorable if, and only if, $\varphi$ is unsatisfiable. Let $\varphi$ be a $\text {HyperLTL}$ formula with quantifier-free part $\psi$. We construct the formula $\varphi '$ from $\varphi$ by replacing the quantifier-free part with $\forall \pi .\exists \pi ' .\psi \rightarrow \Box (a_\pi \rightarrow b_{\pi '})$, where $\pi ,\pi '$ are fresh trace variables and *a*, *b* are fresh atomic propositions. If $\varphi$ is unsatisfiable, the hyperproperty represented by $\varphi '$ is equal to ${\mathcal {P}}(\varSigma ^\omega )$. Thus, every set of prefix traces is good and the formula is monitorable. If $\varphi$ is satisfiable, there exists a set of traces $T \subseteq T^\omega$ such that $T \vDash \varphi$. There are, however, neither good nor bad prefix traces as discussed earlier, i.e., one can duplicate traces in *T* with traces that differ only in the fresh propositions *a* and *b*. Thus, $\varphi '$ is not monitorable. $\square$

*Bounded sequential model* We now consider the case that there is an upper bound $b > 0$ on the number of traces under consideration. We give the adapted definition of monitorability and a characterization for alternation-free $\text {HyperLTL}$. A hyperproperty *H* is *monitorable* in the bounded input model for some bound $b > 0$ if

$$\begin{aligned} \forall U \in {\mathcal {P}}^{\le b}(\varSigma ^*) .\exists V \in {\mathcal {P}}^b(\varSigma ^*) .U \preceq V \wedge (V \in \textit{good}^b(H) \vee V \in \textit{bad}^b(H)) , \end{aligned}$$

where $\textit{good}^b(H) :=\{U \in {\mathcal {P}}^b(\varSigma ^*) \mid \forall V \in {\mathcal {P}}^b(\varSigma ^\omega ) .U \preceq V \Rightarrow V \in H\}$ and $\textit{bad}(H) :=\{U \in {\mathcal {P}}^b(\varSigma ^*) \mid \forall V \in {\mathcal {P}}^b(\varSigma ^\omega ) .U \preceq V \Rightarrow V \notin H\}$, respectively.

#### Theorem 4

Let $\psi$ be the quantifier-free part of some $\forall ^b\text {HyperLTL}$ formula $\varphi$ over trace variables ${\mathcal {V}}$. $\varphi$ is monitorable in the parallel model if, and only if, ${\mathcal {L}}(\psi ) = \varSigma _{\mathcal {V}}^\omega$ or $\forall u \in \varSigma _{\mathcal {V}}^* .\exists v \in \varSigma _{\mathcal {V}}^* .uv \in \textit{bad}({\mathcal {L}}(\psi ))$.

#### Proof

Assume that ${\mathcal {L}}(\psi ) = \varSigma _{\mathcal {V}}^\omega$. It holds that ${\mathcal {H}}(\varphi ) = {\mathcal {P}}(\varSigma ^\omega )$, thus, $\textit{good}^b({\mathcal {H}}(\varphi )) = {\mathcal {P}}^b(\varSigma ^*)$ immediately satisfying the monitorability definition by choosing $V=U$ for every $U \in {\mathcal {P}}^{\le b}(\varSigma ^*)$ if ${|U|} = b$. Otherwise, we start with $V=U$ and add arbitrary traces until ${|V|} = b$.

Assume that $\forall u \in \varSigma _{\mathcal {V}}^* .\exists v \in \varSigma _{\mathcal {V}}^* .uv \in \textit{bad}({\mathcal {L}}(\psi ))$ holds. Let $U \in {\mathcal {P}}^{\le b}(\varSigma ^*)$ be an arbitrary set of prefix traces. Let $U' \in {\mathcal {P}}^b(\varSigma ^*)$ such that $U \preceq U'$ by adding arbitrary traces and such that all traces have the same length by prolonging traces arbitrarily. Let $u' \in \varSigma _{\mathcal {V}}^*$ be a trace corresponding to $U'$ according to the construction in Lemma 2 (there are only finitely many $u'$ corresponding to $U'$ due to different path assignments). By assumption, there is some continuation $v' \in \varSigma _{\mathcal {V}}^*$ of $u'$ such that $u'v' \in \textit{bad}({\mathcal {L}}(\psi ))$. Let *W* be the (canonical) set of prefix traces corresponding to $u'v'$ according to the construction in Lemma 2. By construction, it holds that $U' \preceq W$ and, thus, $U \preceq W$. Furthermore, $W \in \textit{bad}^b({\mathcal {H}}(\varphi ))$ by applying the definitions of $\textit{bad}$ for trace properties and $\textit{bad}^b$ for hyperproperties and translating the traces and trace sets by the construction in Lemma 2.

Assume $\varphi$ is monitorable, thus, $\forall U \in {\mathcal {P}}^{\le b}(\varSigma ^*) .\exists V \in {\mathcal {P}}^b(\varSigma ^*) .U \preceq V \wedge \big ( V \in \textit{good}^b({\mathcal {H}}(\varphi )) \vee V \in \textit{bad}^b({\mathcal {H}}(\varphi )) \big )$. As the set of good prefixes $\textit{good}^b({\mathcal {L}}(\varphi ))$ is empty if ${\mathcal {L}}(\psi ) \ne \varSigma _{\mathcal {V}}^\omega$ by Lemma 3 we can simplify the formula to $\forall U \in {\mathcal {P}}^{\le b}(\varSigma ^*) .\exists V \in {\mathcal {P}}^b(\varSigma ^*) .U \preceq V \wedge V \in \textit{bad}^b({\mathcal {H}}(\varphi ))$ by assuming that ${\mathcal {L}}(\psi ) \ne \varSigma _{\mathcal {V}}^\omega$.

It remains to show that $\forall u \in \varSigma _{\mathcal {V}}^* .\exists v \in \varSigma _{\mathcal {V}}^* .uv \in \textit{bad}({\mathcal {L}}(\psi ))$. Let $u \in \varSigma _{\mathcal {V}}^*$ be arbitrary. We, again, use the translation to the canonical $U' \in {\mathcal {P}}^b(\varSigma ^*)$ from the proof of Lemma 2. There is a continuation $V' \in {\mathcal {P}}^b(\varSigma ^*)$ of $U'$ ($U' \preceq V'$) such that $V' \in \textit{bad}^b({\mathcal {H}}(\varphi ))$ by assumption. By definition of $\preceq$ and the boundedness, it follows that for every $u' \in U'$ there is some $v' \in V'$ such that $u' \preceq v'$. We use this to construct a continuation $v \in \varSigma _{\mathcal {V}}^*$ of *u* such that $uv \in \textit{bad}({\mathcal {L}}(\psi ))$ by using the construction in Lemma 2 again. $\square$

#### Theorem 5

Deciding whether a $\text {HyperLTL}$ formula $\varphi$ is monitorable in the bounded sequential model is undecidable.

#### Proof

As with the unbounded case, we use a reduction from the satisfiability problem of HyperLTL [18]. Given some HyperLTL formula $\varphi$, we construct a formula $\varphi '$ that is monitorable if, and only if, $\varphi$ is unsatisfiable. Since we discussed the special case of alternation-free formulas in Theorem 4, we can assume a formula with quantifier alternations. To do this, we adapt the proof of $\textsc {PSpace}$-hardness for LTL monitorability given in [5]. Let $\varphi$ be a $\text {HyperLTL}$ formula with quantifier-free part $\psi$. We construct the formula $\varphi '$ from $\varphi$ by replacing the quantifier-free part with $\Box a_\pi \vee \Box \diamondsuit (a'_\pi \wedge \psi )$, where $\pi$ is a universal trace variable and $a \in \text {AP}$ and $a'$ is a fresh atomic propositions. If $\varphi$ is unsatisfiable, $\varphi '$ is equivalent to the monitorable LTL formula $\Box a$. If $\varphi$ is satisfiable, there exists a set of traces $T \subseteq T^\omega$ such that $T \vDash \varphi$. It follows that the right part is equivalent to $\Box \diamondsuit a'$ which has neither good nor bad prefixes. Thus, $\varphi '$ is not monitorable. $\square$

*Parallel model* Lastly, we consider the parallel model, were *b* traces are given simultaneously. This model is with respect to monitorability a special case of the bounded model. A hyperproperty *H* is *monitorable* in the fixed size input model if for a given bound *b*

$$\begin{aligned} \forall U \in {\mathcal {P}}^b(\varSigma ^*) .\exists V \in {\mathcal {P}}^b(\varSigma ^*) .U \preceq V \wedge ( V \in \textit{good}^b(H) \vee V \in \textit{bad}^b(H) ). \end{aligned}$$

#### Remark 1

The parallel and bounded sequential model with bound $b = 1$ and a single universal trace quantifier corresponds to the classic monitorability definition for LTL [30].

#### Corollary 5

Let $\psi$ be the quantifier-free part of some $\forall ^b\text {HyperLTL}$ formula $\varphi$ over trace variables ${\mathcal {V}}$. $\varphi$ is monitorable in the parallel model if, and only if, ${\mathcal {L}}(\psi ) = \varSigma _{\mathcal {V}}^\omega$ or $\forall u \in \varSigma _{\mathcal {V}}^* .\exists v \in \varSigma _{\mathcal {V}}^* .uv \in \textit{bad}({\mathcal {L}}(\psi ))$.

#### Proof

A special case of Theorem 4. $\square$

## Monitoring hyperproperties in the parallel model

Whereas the previous section was concerned with the question whether a HyperLTL formula is monitorable, we now focus our attention towards *monitoring algorithms*. As a prerequisite, we recap the semantics of HyperLTL for finite traces [9], which is itself based on the finite trace semantics of LTL [26]. Afterwards, we discuss the monitoring algorithms for the parallel model, where the set of traces is fixed and the traces are processed in parallel, either online in a forward fashion, or offline in a backwards fashion. Figure 1 gives a visual representation how the algorithms process the traces.

### Finite trace semantics

We recap the finite trace semantics for HyperLTL. Let $\varPi _{\textit{fin}}:{\mathcal {V}}\rightarrow \varSigma ^+$ be a partial function mapping trace variables to finite traces. We define $\epsilon [0]$ as the empty set. By slight abuse of notation, we write $t \in \varPi _{\textit{fin}}$ to access traces *t* in the image of $\varPi _{\textit{fin}}$. The satisfaction of a HyperLTL formula $\varphi$ over a finite trace assignment $\varPi _{\textit{fin}}$ and a set of finite traces *T*, denoted by $(T,\varPi _{\textit{fin}},i) \vDash \varphi$, is defined as follows:To enable duality in the finite trace setting, we additionally use the *weak* next operator  and the *release* operator . Due to duality of /, /, $\exists$/$\forall$, and the standard Boolean operators, every HyperLTL formula $\varphi$ can be transformed into negation normal form (NNF) under finite trace semantics, i.e., for every $\varphi$ there is some $\psi$ in negation normal form such that for all $\varPi _{\textit{fin}}$ and *T* it holds that $(T,\varPi _{\textit{fin}},i) \vDash \varphi$ if, and only if, $(T,\varPi _{\textit{fin}},i) \vDash \psi$. We use $\vDash _{\textit{fin}}$ to disambiguate infinite and finite trace semantics where it is not clear from context.

### Monitoring algorithms

In this subsection, we describe our automata-based monitoring algorithm for the parallel model. We present an online algorithm that processes the traces in a forward fashion, and an offline algorithm that processes the traces backwards.

#### Online algorithm

For the online algorithm, we employ standard techniques for building LTL monitoring automata and use this to instantiate this monitor by the traces as specified by the $\text {HyperLTL}$ formula. Let $\text {AP}$ be a set of atomic propositions and ${\mathcal {V}}= \{\pi _1, \ldots , \pi _n\}$ a set of trace variables. A deterministic monitor template ${\mathcal {M}}= (\varSigma , Q, \delta , q_0, F)$ is a tuple of a finite alphabet $\varSigma = {\mathcal {P}}(\text {AP}\times {\mathcal {V}})$, a non-empty set of states *Q*, a partial transition function $\delta : Q \times \varSigma \hookrightarrow Q$, a designated initial state $q_0 \in Q$, and a set of accepting states $F \subseteq Q$. The instantiated automaton runs in parallel over traces in ${\mathcal {P}}(\text {AP})^*$, thus we define a run with respect to a *n*-ary tuple $N \in ({\mathcal {P}}(\text {AP})^*)^n$ of finite traces. A run of *N* is a sequence of states $q_0 q_1 \cdots q_m \in Q^*$, where *m* is the length of the smallest trace in *N*, starting in the initial state $q_0$ such that for all *i* with $0 \le i < m$ it holds that

$$\begin{aligned} \delta \left( q_i, \bigcup _{j=1}^n \bigcup _{a \in N(j)(i)} \{(a,\pi _j)\} \right) = q_{i+1}. \end{aligned}$$

A tuple *N* is accepted, if there is a run on ${\mathcal {M}}$ that ends in an accepting state. For LTL, such a deterministic monitor can be constructed in doubly-exponential time in the size of the formula [14, 35].

##### Example 2

**Fig. 3:**
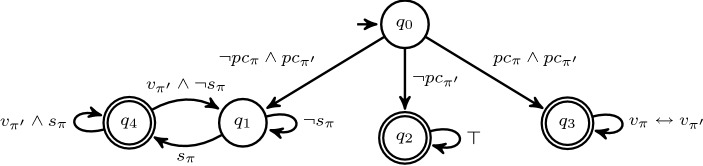
Visualization of a monitor template corresponding to the formula given in Eq. 1. We use a symbolic representation of the transition function $\delta$

We consider again the conference management example from the introduction. We distinguish two types of traces, *author traces* and *program committee member traces*, where the latter starts with proposition *pc*. Based on these traces, we want to verify that no paper submission is lost, i.e., that every submission (proposition *s*) is visible (proposition *v*) to every program committee member in the following step. When comparing two PC traces, we require that they agree on proposition *v*. The monitor template for the following $\text {HyperLTL}$ formalization is depicted in Fig. 3.

1 $$\begin{aligned} \forall \pi . \forall \pi ' .\big ((\lnot pc_\pi \wedge pc_{\pi '}) \rightarrow \Circle \Box (s_\pi \rightarrow \Circle v_{\pi '})\big ) \wedge \big ((pc_{\pi } \wedge pc_{\pi '}) \rightarrow \Circle \Box (v_\pi \leftrightarrow v_\pi ')\big ) \end{aligned}$$

**Fig. 4:**
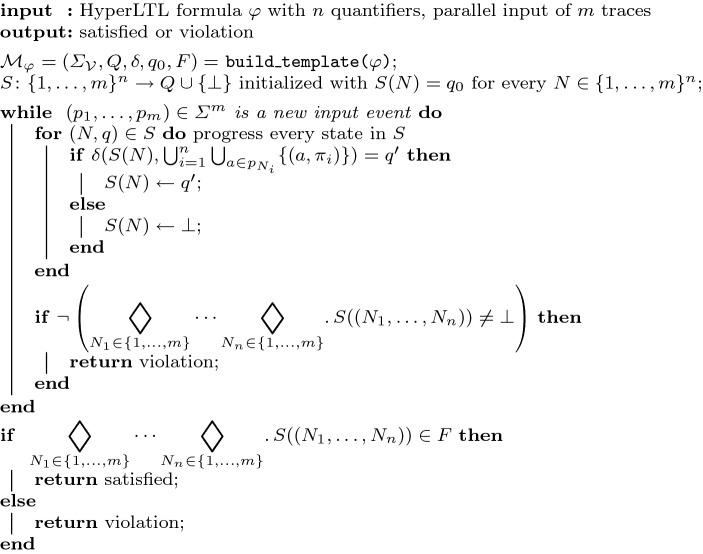
Online algorithm for the parallel model, where $\lozenge _i := \wedge$ if the *i*-th quantifier in $\varphi$ is a universal quantifier and $\vee$ otherwise

The *online* algorithm is depicted in Fig. 4. It proceeds with the pace of the incoming stream. We have a variable *S* that maps tuples of traces to states of the deterministic monitor. Whenever the incoming traces progress, we update the states in *S* according to the transition function $\delta$. If there is a violation on this progress, we return the corresponding tuple of traces as a witness. If the traces ended, the final verdict depends on the current state of the monitor states *S* and the quantifier prefix. Note that because the number of traces is bounded, we can evaluate HyperLTL formulas with arbitrary quantifier prefix.

The space consumption of constructing a deterministic monitor is double-exponential in the size of the formula [14, 35]. Given *m* traces of size *t* and a HyperLTL formula with *n* different quantifiers, the algorithm requires ${\mathcal {O}}(m^n)$ space for maintaining *S* and requires furthermore constant space during runtime. This results in a space consumption of ${\mathcal {O}}( 2^{2^{|\varphi |}} + m^n )$. Each iteration of the loop need ${\mathcal {O}}(m^n)$ time due to the iteration over all elements in *S*, resulting in an overall time complexity of ${\mathcal {O}}( 2^{2^{|\varphi |}} + t \cdot m^n )$, where *t* is the length of the traces.

In the following, we provide the correctness arguments for soundness and completeness of the algorithm. By the construction of the automaton, there are two possibilities for detecting a violation, either by being unable to progress into the next state, or by ending in a non-accepting state, for example, by not fulfilling $\psi$ in $\varphi {\mathcal {U}}\psi$. Both cases are considered by the algorithm by evaluating every trace tuple, i.e., by instantiating the monitor with every *n*-ary trace tuple combination $(N_1,\ldots ,N_n)$. We then build a Boolean combination based on the quantifier prefix, which reduces the correctness of the algorithm to the underlying LTL monitor, as follows: Let $\mathbf {Q}$ denote arbitrary quantifier. By the semantics of HyperLTL, universal quantification over a variable $\pi$ as in $\forall \pi . \mathbf {Q}.\varphi$ requires that for any trace assigned to $\pi$ the formula $\mathbf {Q}.\varphi$ is satisfied, which corresponds to a conjunction over every trace. Dually, existential quantification corresponds to a disjunction.

For the other direction, assume the algorithm reports a violation, i.e., either there is no progress in the monitor or the monitor does not stop in an accepting state. Assume that $T \vDash _{\textit{fin}}\varphi$, thus, there exists a trace mapping $\varPi _{\textit{fin}}$ based on the quantifier prefix, such that $(T,\varPi _{\textit{fin}},0) \vDash _{\textit{fin}}\varphi$. For every satisfying $\varPi _{\textit{fin}}$, there exists an accepting run through ${\mathcal {M}}_\varphi$. This is, by construction, i.e., by emulating the quantification correspondingly, a contradiction to the reporting of a violation.

#### Offline algorithm

For offline analysis, the trace set is completely available, thus, we can avoid constructing the deterministic monitoring automata by using the alternating automaton and a backwards traversal.

An *alternating automaton* [37], whose runs generalize from sequences to trees, is a tuple ${\mathcal {A}} = (\varSigma ,Q,q_0,\delta ,F)$. $\varSigma$ is the alphabet, *Q* is the set of states, $q_0$ is the initial state, and *F* the set of accepting states. $\delta : Q \times \varSigma \rightarrow {\mathbb {B}}^+(Q)$ is a transition function, which maps a state and a symbol into a boolean combination of states. Thus, a run(-tree) of an alternating Büchi automaton ${\mathcal {A}}$ on an infinite word *w* is a *Q*-labeled tree. A word *w* is accepted by ${\mathcal {A}}$ and called a *model* if there exists a run-tree *T* such that all paths *p* through *T* are accepting, i.e., $\mathbf{Inf }(p) \cap F \not = \emptyset$. Note that an alternating automata can be constructed in linear time and space from an LTL formula [37].

**Fig. 5:**
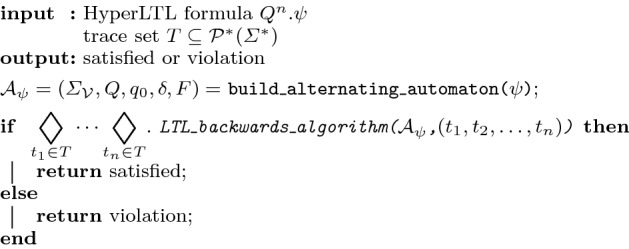
Offline backwards algorithm for the parallel model, where $\lozenge _i := \wedge$ if the *i*-th quantifier in $\varphi$ is a universal quantifier and $\vee$ otherwise

The offline algorithm is depicted in Fig. 5. The input is an arbitrary HyperLTL formula and a trace set *T*. After building the alternating automaton from the quantifier-free part of the HyperLTL formula, we apply the backwards monitoring algorithm [23] for each trace tuple $(t_1,t_2,\ldots ,t_n) \in T^n$, where the quantifier prefix determines satisfaction or violation. The difference to the forward algorithm is that we solely refer to previously computed values, resulting in a more efficient algorithm as shown in the following.

The space consumption of constructing an alternating monitor is linear in the size of the formula. The LTL backwards algorithm requires polynomial memory in the size of the formula, since previously computed values for subformulas have to be stored [23]. This results in a space consumption of ${\mathcal {O}}( {|\varphi |} + {|T|})$. Constructing an alternating automaton requires linear time in the size of the formula. Given *m* traces of size *t* and a HyperLTL formula with *n* different quantifiers, the algorithm runs in ${\mathcal {O}}(m^n \cdot t)$, as the backwards monitoring algorithm is applied for each *n*-ary combination of trace tuples. This results in an overall time consumption of ${\mathcal {O}}( {|\varphi |} + t \cdot m^n )$.

Correctness reduces, with the same arguments as for the online algorithm, to the proven correctness of the backwards algorithm for LTL [23], as the algorithm calls the subprocedure for every trace tuple combination and determines satisfiability according to the quantifier prefix.

##### Remark 2

One important property of runtime verification is the ability to extract *witnesses* from violations. For universal and existential $\text {HyperLTL}$ formulas, the online and offline algorithms can emit traces witnessing violations and satisfactions, respectively.

## Monitoring hyperproperties in the sequential model

After discussing monitoring algorithms for the parallel model in the previous section, we now focus our attention to the case where traces are given sequentially to the monitor. This setting is of interest as it can, for example, detect information flow violations after multiple executions of a program. We also focus on universal HyperLTL formulas, as formulas with alternations are in general not monitorable in this setting (see Sect. 2.2).

**Fig. 6:**
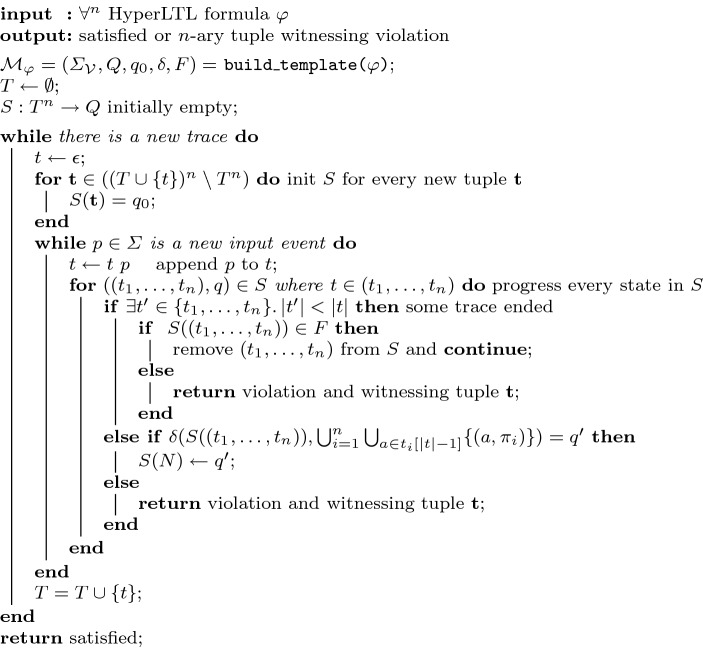
Evaluation algorithm for monitoring $\forall ^n$ HyperLTL formulas in the unbounded sequential model

The algorithm for monitoring $\text {HyperLTL}$ formulas in the unbounded sequential model is presented in Fig. 6. After building the deterministic monitoring automaton ${\mathcal {M}}_\varphi$, the algorithm accepts new traces and afterwards proceeds with the pace of the incoming stream. We have a variable *S* that maps tuples of traces to states of the deterministic monitor. Whenever the current trace *t* progresses, we progress every tuple $(t_1,\ldots ,t_n)$ that contains *t* with one of the following outcomes:

1. One of the traces $t_1,\ldots ,t_n$ may have ended, thus, we check if the monitor is in an accepting state and report a violation if this is not the case.
2. There is a successor state in the monitor, thus we update *S*.
3. There is no successor state, hence, we report a violation.

When a new trace *t* starts, only new tuples are considered for *S*, that are tuples $\mathbf {t} \in (T \cup \{t\})^n$ containing the new trace *t*.

Given a HyperLTL formula with *n* different quantifiers, the algorithm requires ${\mathcal {O}}({|T|}^n)$ space during runtime. This results in a space consumption of ${\mathcal {O}}( 2^{2^{|\varphi |}} + {|T|}^n )$. For *m* traces of length *t*, the algorithm requires ${\mathcal {O}}(m^n)$ time for each iteration, resulting in an overall time complexity of ${\mathcal {O}}( 2^{2^{|\varphi |}} + t \cdot m^n )$.

The correctness arguments for the parallel model translate to the sequential model: since we solely consider $\forall ^n$ HyperLTL formulas, it is sufficient, by the finite trace semantics of HyperLTL, to check every combination of trace tuples $(t_1, \ldots , t_n)$ and immediately report a violation if the automaton cannot progress or stops in a non-accepting state.

*Bounded model* In the bounded model, we have an upper bound *m* on the number of traces. Thus, we can update the algorithm presented in Fig. 6 to evaluate an arbitrary quantifier prefix similar to the algorithms in Figs. 4 and 5. However, in general, we lose the ability to detect violations and satisfactions during monitoring as we have to reach the bound *m* to be able to compute a verdict.

A benefit to the monitoring algorithm presented in this section is that it is able to return a witness for violation. As a negative consequence, we have to remember each and every trace seen so far. This is, in general, unavoidable, as shown by the following hyperproperty: “every new trace has to be different than the one before”. However, it turns out that in many cases hyperproperties satisfy certain properties that allows us to prune a majority of traces during the monitoring process. We discuss these cases in the following.

### Optimizations

As discussed in the introduction, the main obstacle in monitoring hyperproperties in the unbounded input model is the potentially unbounded space consumption. In the following, we present two analysis phases of our algorithm. The first phase is a specification analysis, which is a preprocessing step that analyzes the HyperLTL formula under consideration. We use the recently introduced satisfiability solver for hyperproperties EAHyper [19] to detect whether a formula is (1) *symmetric*, i.e., we halve the number of instantiated monitors, (2) *transitive*, i.e, we reduce the number of instantiated monitors to two, or (3) *reflexive*, i.e., we can omit the self comparison of traces. The second analysis phase is applied during runtime. We analyze the incoming trace, with respect to a given HyperLTL formula, to detect whether this trace poses strictly less requirements on future traces. If so, the trace does not need to be stored.

#### Specification analysis

*Symmetry* We call a HyperLTL formula symmetric if permuting the trace variables does not alter the satisfaction of the formula, which is especially interesting since many information flow policies satisfy this property. Consider, for example, observational determinism $\forall \pi .\forall \pi '.(O_\pi = O_{\pi '}) {\mathcal {W}}(I_\pi \ne I_{\pi '}).$ We detect symmetry by translating this formula to a formula that is unsatisfiable if there exists no set of traces for which every trace pair violates the symmetry condition: $\exists \pi .\exists \pi '.\big ((O_\pi = O_{\pi '}) {\mathcal {W}}(I_\pi \ne I_{\pi '})\big ) \nleftrightarrow \big ((O_\pi ' = O_{\pi }) {\mathcal {W}}(I_\pi ' \ne I_{\pi })\big ).$ This is a sufficient condition for the invariance under the trace variable permutation of $\pi$ and $\pi '$, which we define in the following, and, therefore, observational determinism is symmetric.

##### Definition 1

Let $\psi$ be the quantifier-free part of some $\text {HyperLTL}$ formula $\varphi$ over trace variables ${\mathcal {V}}$. We say $\varphi$ is invariant under trace variable permutation $\sigma : {\mathcal {V}}\rightarrow {\mathcal {V}}$, if for any set of traces $T \subseteq \varSigma ^{\omega }$ and any assignment $\varPi : {\mathcal {V}}\rightarrow T$, $(\emptyset , \varPi , 0) \vDash \psi \Leftrightarrow (\emptyset , \varPi \circ \sigma , 0) \vDash \psi$. We say $\varphi$ is symmetric, if it is invariant under every trace variable permutation in ${\mathcal {V}}$.

We generalize the previous example to formulas with more than two universal quantifiers. We use the fact, that the symmetric group for a finite set ${\mathcal {V}}$ of *n* trace variables is generated by the two permutations $(\pi _1 \; \pi _2)$ and $(\pi _1 \; \pi _2 \; \cdots \; \pi _{n-1} \; \pi _n)$. If the $\text {HyperLTL}$-SAT solver determines that the input formula is invariant under these two permutations, then the formula is invariant under every trace variable permutation and thus symmetric. We use the notation $\psi (\pi _{j_1},\ldots ,\pi _{j_n})$ to denote the altered formula $\psi$, where every occurrence of $\pi _i$ is replaced with $\pi _{j_i}$.

##### Theorem 6

Let $$\psi $$ be the quantifier-free part of some $$\text {HyperLTL}$$ formula $$\varphi $$ over trace variables $${\mathcal {V}}= \{\pi _1, \ldots , \pi _n\}$$. $$\varphi $$ is symmetric if and only if $$ \varphi _{\textit{symm}} = \exists \pi _1 \ldots \exists \pi _n .(\psi (\pi _1, \pi _2, \ldots , \pi _{n-1}, \pi _n) \nleftrightarrow \psi (\pi _2, \pi _1, \ldots , \pi _{n-1}, \pi _n)) $$$$ \vee (\psi (\pi _1, \pi _2, \ldots , \pi _{n-1}, \pi _n) \nleftrightarrow \psi (\pi _2, \pi _3, \ldots , \pi _n, \pi _1)) $$ is unsatisfiable.

##### Proof

Note for any trace variable permutation $\sigma : {\mathcal {V}}\rightarrow {\mathcal {V}}$, any set of traces $T \in \varSigma ^\omega$ and any trace assignment $\varPi : {\mathcal {V}}\rightarrow T$ the following holds: $(\emptyset , \varPi , 0) \vDash \psi \Leftrightarrow (\emptyset , \varPi \circ \sigma , 0) \vDash \psi (\sigma (\pi _1), \sigma (\pi _2), \ldots , \sigma (\pi _{n-1}), \sigma (\pi _n))$.

Let $\varphi$ be a symmetric HyperLTL formula with quantifier-free part $\psi$, i.e., by Definition 1 it is invariant under both trace variable permutations $\sigma _{\textit{swap}} = (\pi _1 \; \pi _2)$ and $\sigma _{\textit{rot}} = (\pi _1 \; \pi _2 \; \cdots \; \pi _{n-1} \; \pi _n)$, i.e., for any set of traces $T \in \varSigma ^\omega$ and any trace assignment $\varPi : {\mathcal {V}}\rightarrow T$, $(\emptyset , \varPi , 0) \vDash \psi \Leftrightarrow (\emptyset , \varPi \circ \sigma _{\textit{swap}}, 0) \vDash \psi$ and $(\emptyset , \varPi , 0) \vDash \psi \Leftrightarrow (\emptyset , \varPi \circ \sigma _{\textit{rot}}, 0) \vDash \psi$ hold. So there is neither a trace assignment satisfying ${(\psi (\pi _1, \pi _2, \ldots , \pi _{n-1}, \pi _n) \nleftrightarrow \psi (\pi _2, \pi _1, \ldots , \pi _{n-1}, \pi _n))}$ nor satisfying ${(\psi (\pi _1, \pi _2, \ldots , \pi _{n-1}, \pi _n) \nleftrightarrow \psi (\pi _2, \pi _3, \dots , \pi _n, \pi _1))}$. Therefore $\varphi _{symm}$ is unsatisfiable.

For the other direction assume $\varphi _{symm}$ is unsatisfiable. This implies that for any set of traces $T \in \varSigma ^\omega$ and any trace assignment $\varPi : {\mathcal {V}}\rightarrow T$, $(\emptyset , \varPi , 0) \vDash \psi \Leftrightarrow (\emptyset , \varPi \circ \sigma _{\textit{swap}}, 0) \vDash \psi$ and $(\emptyset , \varPi , 0) \vDash \psi \Leftrightarrow (\emptyset , \varPi \circ \sigma _{\textit{rot}}, 0) \vDash \psi$ hold. Let $\sigma : {\mathcal {V}}\rightarrow {\mathcal {V}}$ be an arbitrary trace permutation over ${\mathcal {V}}$. By the fact that $\sigma$ can get represented as a finite composition of $\sigma _{\textit{swap}}$ and $\sigma _{\textit{rot}}$, i.e., $\sigma = \sigma _1 \circ \cdots \circ \sigma _k$ for $\sigma _i \in \{\sigma _{\textit{swap}}, \sigma _{\textit{rot}}\}$, we can conclude the proof with the following construction. Let $T \in \varSigma ^\omega$ be an arbitrary set of traces and $\varPi : {\mathcal {V}}\rightarrow T$ be an arbitrary trace assignment. Define $\varPi _0 := \varPi$, $\varPi _i := \varPi _{i-1} \circ \sigma _i$. Then we get $(\emptyset , \varPi , 0) \vDash \psi \Leftrightarrow (\emptyset , \varPi _1, 0) \vDash \psi \Leftrightarrow \cdots \Leftrightarrow (\emptyset , \varPi _k, 0) = (\emptyset , \varPi \circ \sigma , 0) \vDash \psi$. Therefore $\psi$ is invariant under the trace variable permutation $\sigma$. Since $\sigma$ was chosen arbitrarily, $\varphi$ is symmetric. $\square$

*Transitivity* While symmetric HyperLTL formulas allow us to prune half of the monitor instances, transitivity of a HyperLTL formula has an even larger impact on the required memory. Observational determinism, considered above, is not transitive. However, equality, i.e, $\forall \pi . \forall \pi ' .\Box (a_\pi \leftrightarrow a_{\pi '})$, for example, is transitive and symmetric and allows us to reduce the number of monitor instances to one, since we can check equality against any reference trace.

##### Definition 2

Let $\psi$ be the quantifier-free part of some $\text {HyperLTL}$ formula $\varphi$ over trace variables $\{\pi _1, \pi _2\}$. Let $T = \{t_1, t_2, t_3\} \in \varSigma ^{\omega }$ be a three-elemented set of traces. We define the assignment $\varPi _{i,j} : {\mathcal {V}}\rightarrow \varSigma ^{\omega }$ by $\varPi _{i,j} :=\{\pi _1 \mapsto t_i, \pi _2 \mapsto t_j\}$. We say $\varphi$ is transitive, if *T* was chosen arbitrarily and $(\emptyset , \varPi _{1,2}, 0) \vDash \psi \wedge (\emptyset , \varPi _{2,3}, 0) \vDash \psi \rightarrow (\emptyset , \varPi _{1,3}, 0) \vDash \psi$.

##### Theorem 7

Let $\psi$ be the quantifier-free part of some $\text {HyperLTL}$ formula $\varphi$ over trace variables $\{\pi _1, \pi _2\}$. $\varphi$ is transitive if and only if $\varphi _{\textit{trans}} = \exists \pi _1 \exists \pi _2 \exists \pi _3 .(\psi (\pi _1, \pi _2) \wedge \psi (\pi _2, \pi _3)) \nrightarrow \psi (\pi _1, \pi _3)$ is unsatisfiable.

##### Proof

Let *T* be an arbitrary three-elemented set of traces and let $\varphi$ be a transitive HyperLTL formula over trace variables $\{\pi _1,\pi _2\}$ with quantifier-free part $\psi$, i.e., $(\emptyset , \varPi _{1,2}, 0) \vDash \psi \wedge (\emptyset , \varPi _{2,3}, 0) \vDash \psi \rightarrow (\emptyset , \varPi _{1,3}, 0) \vDash \psi$ by Definition 2. Equivalently, there does not exists any tree-elemented set, such that $(\emptyset , \varPi _{1,2}, 0) \vDash \psi \wedge (\emptyset , \varPi _{2,3}, 0) \vDash \psi \not \rightarrow (\emptyset , \varPi _{1,3}, 0) \vDash \psi$. This is semantically equivalent to $\exists \pi _1 \exists \pi _2 \exists \pi _3 .(\psi (\pi _1, \pi _2) \wedge \psi (\pi _2, \pi _3)) \nrightarrow \psi (\pi _1, \pi _3) = \varphi _{\textit{trans}}$ being unsatisfiable. $\square$

*Reflexivity* Lastly, we introduce a method to check whether a formula is reflexive, which enables us to omit the composition of a trace with itself in the monitoring algorithm. For example, equality and observational determinism have reflexive HyperLTL formulas.

##### Definition 3

Let $\psi$ be the quantifier-free part of some $\text {HyperLTL}$ formula $\varphi$ over trace variables ${\mathcal {V}}$. We say $\varphi$ is reflexive, if for any trace $t \in \varSigma ^{\omega }$ and the corresponding assignment $\varPi : {\mathcal {V}}\rightarrow \{t\}$, $(\emptyset , \varPi , 0) \vDash \psi$.

##### Theorem 8

Let $\psi$ be the quantifier-free part of some $\text {HyperLTL}$ formula $\varphi$ over trace variables ${\mathcal {V}}$. $\varphi$ is reflexive if and only if $\varphi _{\textit{refl}} = \exists \pi .\lnot \psi (\pi , \pi , \ldots , \pi )$ is unsatisfiable.

##### Proof

Let $\varphi$ be a reflexive HyperLTL formula with quantifier-free part $\psi$, i.e., for any trace $t \in \varSigma ^\omega$ with trace assignment $\varPi : {\mathcal {V}}\rightarrow \{t\}$ it holds that $(\emptyset , \varPi , 0) \vDash \psi$ by Definition 3. Equivalently there does not exist any trace $t \in \varSigma ^\omega$ with trace assignment $\varPi : {\mathcal {V}}\rightarrow \{t\}$ such that $(\emptyset , \varPi , 0) \nvDash \psi$ holds. This is semantically equivalent to $\exists \pi . \lnot \psi (\pi ,\pi , \ldots , \pi ) = \varphi _{\textit{refl}}$ being unsatisfiable. $\square$

#### Trace analysis

In the previous subsection, we described a preprocessing step to reduce the number of monitor instantiations. The main idea of the trace analysis, considered in the following, is to check whether a trace contains new requirements on the system under consideration. If this is not the case, then this trace will not be stored by our monitoring algorithm. In this section, we use the standard automaton construction for universal HyperLTL [12] to construct a Büchi automaton ${\mathcal {A}}_\varphi$ that accepts zipped traces, i.e., traces in $\varSigma _{\mathcal {V}}$. We denote the (trace) language of the automaton by ${\mathcal {L}}({\mathcal {A}}_\varphi )$. For a given HyperLTL formula $\varphi$, a trace $t \in \varSigma ^\omega$ and an automaton ${\mathcal {A}}$ over ${\mathcal {V}}= \{\pi _1, \ldots , \pi _n\}$, we define ${\mathcal {A}}_\varphi [t/\pi _i]$ as the automaton over ${\mathcal {V}}= \{\pi _1, \ldots , \pi _n\} \backslash \{\pi _i\}$, where the transitions for $\pi _i$ are fixed according to *t*. We call this operation the *initialization* of $\pi$ with *t*. Note that if $n = 1$, i.e., $n - 1$ traces have been initialized, the automaton is a Büchi automaton over a trace variable.

##### Definition 4

Given a HyperLTL formula $\varphi$, a trace set *T* and an arbitrary $t \in \varSigma ^\omega$, we say that *t* is $(T,\varphi )$-redundant if *T* is a model of $\varphi$ if and only if $T \cup \{t\}$ is a model of $\varphi$ as well, formally

$$\begin{aligned} \forall T' \supseteq T .T' \in {\mathcal {H}}(\varphi ) \Leftrightarrow T' \cup \{t\} \in {\mathcal {H}}(\varphi ) . \end{aligned}$$

##### Example 3

Consider, again, our example hyperproperty for a conference management system. *“A user submission is immediately visible for every program committee memberand every program committee member observes the same.”* We formalized this property as a $\forall ^2$ HyperLTL formula in Eq. 1. Assume our algorithm observes the following three traces of length five. 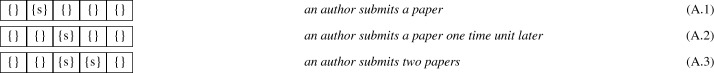


Trace A.2 contains, with respect to $\varphi$ above, no more information than trace A.3. We say that trace A.3 dominates trace A.2 and, hence, trace A.2 may be pruned from the set of traces that the algorithm has to store. If we consider a PC member trace, we encounter the following situation. 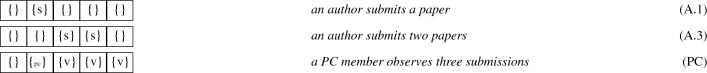
Our algorithm will detect no violation, since the program committee member sees all three papers. Intuitively, one might expect that no more traces can be pruned from this trace set. In fact, trace PC dominates trace A.1 and trace A.3: the information that three papers have been submitted is implied by the observation in trace PC, since a part of property states for all traces $\pi ,\pi '$ that . Hence, it suffices to remember the last trace to detect, for example, the following violations. 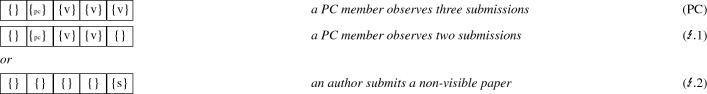 Note that none of the previous user traces, i.e., trace A.1 to trace A.3, are needed to detect a violation.

##### Definition 5

Given $t,t'\in \varSigma ^\omega$, we say *tdominates*$t'$ with respect to $\varphi$ (or simply *tdominates*$t'$ if it is clear from the context) if $t'$ is $(\{t\},\varphi )$-redundant.

The observations from Example 3 can be generalized to a language inclusion check (cf. Theorem 9), to determine whether a trace dominates another trace. For proving this, we first prove the following two lemmas. We start by considering $\forall ^2\text {HyperLTL}$ formulas.

##### Lemma 4

Let $\varphi$ be a $\forall ^2\text {HyperLTL}$ formula over trace variables $\{\pi _1, \pi _2\}$. Given an arbitrary trace set *T* and an arbitrary trace *t* satisfying $\varphi$, i.e., $T,\{t\} \in {\mathcal {H}}(\varphi )$. $T \cup \{t\}$ as well is a model of $\varphi$ if and only if *T* is accepted by the two automata where once $\pi _1$ is initialized with *t* and once $\pi _2$ is initialized with *t*. Formally, the following equivalence holds:

$$\begin{aligned} \forall T,\{t\} \in {\mathcal {H}}(\varphi ) .T \cup \{t\} \in {\mathcal {H}}(\varphi ) \Leftrightarrow T \subseteq {\mathcal {L}}({\mathcal {A}}_\varphi [t/\pi _1]) \wedge T \subseteq {\mathcal {L}}({\mathcal {A}}_\varphi [t/\pi _2]) \end{aligned}$$

##### Proof

Lets say $\varphi$ is of the form $\forall \pi _1 \forall \pi _2 .\psi$. According to the semantics of $\text {HyperLTL}$, the set $T \cup \{t\}$ satisfies $\varphi$ if and only if each trace assignment $\varPi = \{\pi _1 \mapsto t_1, \pi _2 \mapsto t_2\}$ with $t_1,t_2 \in T \cup \{t\}$ satisfies $\psi$. As $T,\{t\} \in {\mathcal {H}}(\varphi )$, we already know that this holds if $t_1$ and $t_2$ are solely chosen from *T* and also $\{\pi _1 \mapsto t, \pi _2 \mapsto t\}$ satisfies $\psi$. This leaves us with trace assignments $\{\pi _1 \mapsto t, \pi _2 \mapsto t'\}$, $\{\pi _1 \mapsto t', \pi _2 \mapsto t\}$ where $t' \in T$. By the definition of ${\mathcal {A}}_\varphi$, ${\mathcal {L}}({\mathcal {A}}_\varphi [t/\pi _1])$ contains exactly those traces $t'$ for which the corresponding trace assignment $\{\pi _1 \mapsto t, \pi _2 \mapsto t'\}$ satisfies $\psi$. Respectively ${\mathcal {L}}({\mathcal {A}}_\varphi [t/\pi _2])$ contains the traces $t'$ corresponding to satisfying assignments $\{\pi _1 \mapsto t', \pi _2 \mapsto t\}$. This concludes that $T \cup \{t\} \in {\mathcal {H}}(\varphi )$ if and only if $T \subseteq {\mathcal {L}}({\mathcal {A}}_\varphi [t/\pi _1])$ and $T \subseteq {\mathcal {L}}({\mathcal {A}}_\varphi [t/\pi _2])$. $\square$

##### Corollary 6

Let $\varphi$ be an $\exists ^2\text {HyperLTL}$ formula over trace variables $\{\pi _1, \pi _2\}$. Given an arbitrary trace set *T* and an arbitrary trace *t* not satisfying $\varphi$, i.e., $T,\{t\} \notin {\mathcal {H}}(\varphi )$. $T \cup \{t\}$ as well is not a model of $\varphi$ if and only if the intersection of *T* and any of the languages of the two automata, where once $\pi _1$ is initialized with *t* and once $\pi _2$ is initialized with *t*, is empty. Formally, the following equivalence holds:

$$\begin{aligned} \forall T,\{t\} \notin {\mathcal {H}}(\varphi ) .T \cup \{t\} \notin {\mathcal {H}}(\varphi ) \Leftrightarrow T \cap {\mathcal {L}}({\mathcal {A}}_\varphi [t/\pi _1]) = \emptyset = T \cap {\mathcal {L}}({\mathcal {A}}_\varphi [t/\pi _2]) \end{aligned}$$

##### Proof

Lets say $\varphi$ is of the form $\exists \pi _1 \exists \pi _2 .\psi$. According to the semantics of $\text {HyperLTL}$, the set $T \cup \{t\}$ does not satisfy $\varphi$ if and only if each trace assignment $\varPi = \{\pi _1 \mapsto t_1, \pi _2 \mapsto t_2\}$ with $t_1,t_2 \in T \cup \{t\}$ does not satisfy $\psi$. As $T,\{t\} \notin {\mathcal {H}}(\varphi )$, we already know that this holds if $t_1$ and $t_2$ are solely chosen from *T* and also $\{\pi _1 \mapsto t, \pi _2 \mapsto t\}$ does not satisfy $\psi$. This leaves us with trace assignments $\{\pi _1 \mapsto t, \pi _2 \mapsto t'\}$, $\{\pi _1 \mapsto t', \pi _2 \mapsto t\}$ where $t' \in T$. By the definition of ${\mathcal {A}}_\varphi$, ${\mathcal {L}}({\mathcal {A}}_\varphi [t/\pi _1])$ contains exactly those traces $t'$ for which the corresponding trace assignment $\{\pi _1 \mapsto t, \pi _2 \mapsto t'\}$ satisfies $\psi$. Respectively ${\mathcal {L}}({\mathcal {A}}_\varphi [t/\pi _2])$ contains the traces $t'$ corresponding to satisfying assignments $\{\pi _1 \mapsto t', \pi _2 \mapsto t\}$. This concludes that $T \cup \{t\} \notin {\mathcal {H}}(\varphi )$ if and only if $T \cap {\mathcal {L}}({\mathcal {A}}_\varphi [t/\pi _1]) = \emptyset$ and $T \cap {\mathcal {L}}({\mathcal {A}}_\varphi [t/\pi _2]) = \emptyset$. $\square$

##### Lemma 5

Given a $\forall ^2\text {HyperLTL}$ formula $\varphi$ over trace variables $\{\pi _1, \pi _2\}$ and two traces $t,t' \in \varSigma ^\omega$ with $\{t'\} \in {\mathcal {H}}(\varphi )$. The following holds: *t* dominates $t'$ if

$$\begin{aligned} {\mathcal {L}}({\mathcal {A}}_\varphi [t/\pi _1]) \subseteq {\mathcal {L}}({\mathcal {A}}_\varphi [t'/\pi _1]) \text { and } {\mathcal {L}}({\mathcal {A}}_\varphi [t/\pi _2]) \subseteq {\mathcal {L}}({\mathcal {A}}_\varphi [t'/\pi _2]) \end{aligned}$$

##### Proof

Assume that ${\mathcal {L}}({\mathcal {A}}_\varphi [t/\pi _1]) \subseteq {\mathcal {L}}({\mathcal {A}}_\varphi [t'/\pi _1])$ and ${\mathcal {L}}({\mathcal {A}}_\varphi [t/\pi _2]) \subseteq {\mathcal {L}}({\mathcal {A}}_\varphi [t'/\pi _2])$ holds. Let *T* be an arbitrary trace set with $T \supseteq \{t\}$. We distinguish two cases:

- Case $T \in {\mathcal {H}}(\varphi )$, then ${T \subseteq {\mathcal {L}}({\mathcal {A}}_\varphi [t/\pi _1]) \subseteq {\mathcal {L}}({\mathcal {A}}_\varphi [t'/\pi _1])}$ and $T \subseteq {\mathcal {L}}({\mathcal {A}}_\varphi [t/\pi _2]) \subseteq {\mathcal {L}}({\mathcal {A}}_\varphi [t'/\pi _2])$. By Lemma 4 it follows that $T \cup \{t'\} \in {\mathcal {H}}(\varphi )$.
- Case $T \notin {\mathcal {H}}(\varphi )$, then $T \cup \{{\tilde{t}}\} \notin {\mathcal {H}}(\varphi )$ for any trace ${\tilde{t}}$. In particular $T \cup \{t'\} \notin {\mathcal {H}}(\varphi )$.

This meets Definition 5, therefore *t* dominates $t'$. $\square$

**Fig. 7:**
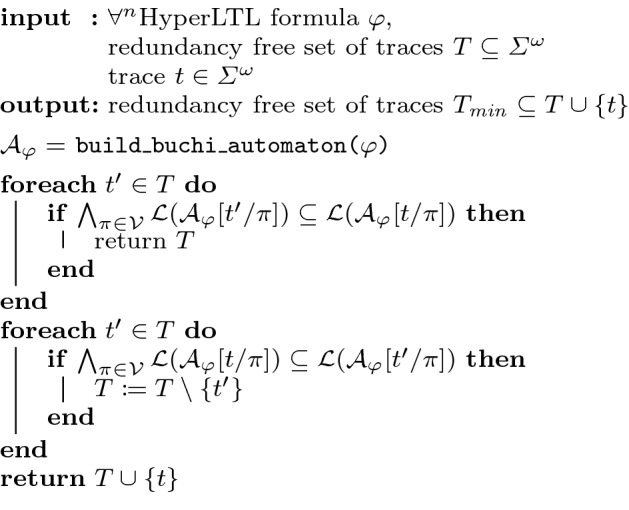
Storage Minimization Algorithm

A generalization leads to the following theorem, which serves as the foundation of our trace storage minimization algorithm depicted in Fig. 7.

##### Theorem 9

Given a $\forall ^n\text {HyperLTL}$ formula $\varphi$ over trace variables ${\mathcal {V}}= \{\pi _1, \ldots , \pi _n\}$ and two traces $t,t' \in \varSigma ^\omega$ with $\{t'\} \in {\mathcal {H}}(\varphi )$. The following holds: *t* dominates $t'$ if

$$\begin{aligned} \bigwedge _{\pi \in {\mathcal {V}}}{\mathcal {L}}({\mathcal {A}}_\varphi [t/\pi ]) \subseteq {\mathcal {L}}({\mathcal {A}}_\varphi [t'/\pi ]) . \end{aligned}$$

The characterization of dominance for existential quantification is dual.

##### Lemma 6

Given an $\exists ^2$ HyperLTL formula $\varphi$ over trace variables ${\mathcal {V}}= \{\pi _1, \pi _2\}$ and two traces $t,t' \in \varSigma ^\omega$ with $\{t'\} \notin {\mathcal {H}}(\varphi )$. The following holds: *t* dominates $t'$ if

$$\begin{aligned} {\mathcal {L}}({\mathcal {A}}_\varphi [t'/\pi _1]) \subseteq {\mathcal {L}}({\mathcal {A}}_\varphi [t/\pi _1]) \text { and } {\mathcal {L}}({\mathcal {A}}_\varphi [t'/\pi _2]) \subseteq {\mathcal {L}}({\mathcal {A}}_\varphi [t/\pi _2]) \end{aligned}$$

##### Proof

Assume that ${\mathcal {L}}({\mathcal {A}}_\varphi [t'/\pi _1]) \subseteq {\mathcal {L}}({\mathcal {A}}_\varphi [t/\pi _1])$ and ${\mathcal {L}}({\mathcal {A}}_\varphi [t'/\pi _2]) \subseteq {\mathcal {L}}({\mathcal {A}}_\varphi [t/\pi _2])$ holds. Let *T* be an arbitrary trace set with $T \supseteq \{t\}$. We distinguish two cases:

- Case $T \notin {\mathcal {H}}(\varphi )$, then ${T \cap {\mathcal {L}}({\mathcal {A}}_\varphi [t/\pi _1]) = \emptyset }$ and ${T \cap {\mathcal {L}}({\mathcal {A}}_\varphi [t/\pi _2]) = \emptyset }$. Therefore also ${T \cap {\mathcal {L}}({\mathcal {A}}_\varphi [t'/\pi _1]) = \emptyset }$ and ${T \cap {\mathcal {L}}({\mathcal {A}}_\varphi [t'/\pi _2]) = \emptyset }$. By Corollary 6, it follows that $T \cup \{t'\} \notin {\mathcal {H}}(\varphi )$.
- Case $T \in {\mathcal {H}}(\varphi )$, then $T \cup \{{\tilde{t}}\} \in {\mathcal {H}}(\varphi )$ for any trace ${\tilde{t}}$. In particular $T \cup \{t'\} \in {\mathcal {H}}(\varphi )$.

This meets Definition 5, therefore *t* dominates $t'$. $\square$

##### Corollary 7

Given an $\exists ^n\text {HyperLTL}$ formula $\varphi$ over trace variables ${\mathcal {V}}= \{\pi _1, \ldots , \pi _n\}$ and two traces $t,t' \in \varSigma ^\omega$ with $\{t'\} \notin {\mathcal {H}}(\varphi )$. The following holds: *t* dominates $t'$ if $\bigwedge _{\pi \in {\mathcal {V}}}{\mathcal {L}}({\mathcal {A}}_\varphi [t'/\pi ]) \subseteq {\mathcal {L}}({\mathcal {A}}_\varphi [t/\pi ])$.

##### Theorem 10

Algorithm 7 preserves the minimal trace set *T*, i.e., for all $t \in T$ it holds that *t* is not $(T {\setminus } \{t\},\varphi )$-redundant.

##### Proof

By induction on $T {\setminus } \{t\}$ and Theorem 9. $\square$

In the following, we give a characterization of the trace dominance for $\text {HyperLTL}$ formulas with one alternation. These characterizations can be checked similarly to the algorithm depicted in Fig. 7.

##### Theorem 11

Given a $\text {HyperLTL}$ formula $\varphi = \forall \pi .\exists \pi ' .\psi$ two traces $t,t' \in \varSigma ^\omega$, the following holds: *t* dominates $t'$ if

$$\begin{aligned} {\mathcal {L}}({\mathcal {A}}_\varphi [t/\pi ]) \subseteq {\mathcal {L}}({\mathcal {A}}_\varphi [t'/\pi ]) \text { and } {\mathcal {L}}({\mathcal {A}}_\varphi [t'/\pi ']) \subseteq {\mathcal {L}}({\mathcal {A}}_\varphi [t/\pi ']) . \end{aligned}$$

##### Proof

Assume that (a) ${\mathcal {L}}({\mathcal {A}}_\varphi [t/\pi ]) \subseteq {\mathcal {L}}({\mathcal {A}}_\varphi [t'/\pi ])$ and (b) ${\mathcal {L}}({\mathcal {A}}_\varphi [t'/\pi ']) \subseteq {\mathcal {L}}({\mathcal {A}}_\varphi [t/\pi '])$ hold. Let *T* be arbitrary such that $\{t\} \subseteq T$. We distinguish two cases:

- Case $T \in {\mathcal {H}}(\varphi )$, then for all $t_1 \in T$ there is a $t_2 \in T$ such that $(\emptyset , \{\pi \mapsto t_1, \pi ' \mapsto t_2\}, 0) \vDash \psi$. Especially, for *t*, there is a corresponding trace ${\tilde{t}}$ such that $(\emptyset , \{\pi \mapsto t, \pi ' \mapsto {\tilde{t}}\}, 0) \vDash \psi$, thus ${\tilde{t}} \in {\mathcal {L}}({\mathcal {A}}_\varphi [t/\pi ])$. From (a) it follows that ${\tilde{t}} \in {\mathcal {L}}({\mathcal {A}}_\varphi [t'/\pi ])$ as well. Hence, $(\emptyset , \{\pi \mapsto t', \pi ' \mapsto {\tilde{t}}\}, 0) \vDash \psi$ and thereby $T \cup \{t'\} \in {\mathcal {H}}(\varphi )$.
- Case $$T \notin {\mathcal {H}}(\varphi )$$, then there exists a $${\tilde{t}} \in T$$ such that for all $${\hat{t}} \in T$$$$(\emptyset , \{\pi \mapsto {\tilde{t}}, \pi ' \mapsto {\hat{t}}\}, 0) \nvDash \psi $$ holds. Especially, for *t*, $$(\emptyset , \{\pi \mapsto {\tilde{t}}, \pi ' \mapsto t\}, 0) \nvDash \psi $$, thus $${\tilde{t}} \notin {\mathcal {L}}({\mathcal {A}}_\varphi [t/\pi '])$$. From (b) it follows that $${\tilde{t}} \notin {\mathcal {L}}({\mathcal {A}}_\varphi [t'/\pi '])$$ as well. Hence, $$(\emptyset , \{\pi \mapsto {\tilde{t}}, \pi ' \mapsto t'\}, 0) \nvDash \psi $$. As there is no trace $${\hat{t}} \in T \cup \{t'\}$$ such that $$(\emptyset , \{\pi \mapsto {\tilde{t}}, \pi ' \mapsto {\hat{t}}\}, 0) \vDash \psi $$, we can conclude $$T \cup \{t'\} \notin {\mathcal {H}}(\varphi )$$. $$\square $$

##### Corollary 8

Given a $\text {HyperLTL}$ formula $\varphi = \exists \pi .\forall \pi ' .\psi$ two traces $t,t' \in \varSigma ^\omega$, the following holds: *t* dominates $t'$ if and only if

$$\begin{aligned} {\mathcal {L}}({\mathcal {A}}_\varphi [t'/\pi ]) \subseteq {\mathcal {L}}({\mathcal {A}}_\varphi [t/\pi ]) \text { and } {\mathcal {L}}({\mathcal {A}}_\varphi [t/\pi ']) \subseteq {\mathcal {L}}({\mathcal {A}}_\varphi [t'/\pi ']) . \end{aligned}$$

##### Example 4

We show the effect of the dominance characterization on two example formulas. Consider the $\text {HyperLTL}$ formula $\forall \pi .\exists \pi ' .\Box (a_\pi \rightarrow b_{\pi '})$ and the traces $\{b\} \emptyset$, $\{b\}\{b\}$, $\{a\} \emptyset$, and $\{a\}\{a\}$. Trace $\{a\}\{a\}$ dominates trace $\{a\}\emptyset$ as instantiating $\pi$ requires two consecutive *b*’s for $\pi '$ where $\{a\}\emptyset$ only requires a *b* at the first position (both traces do not contain *b*’s, so instantiating $\pi '$ leads to the same language). Similarly, one can verify that $\{b\}\{b\}$ dominates trace $\{b\}\emptyset$.

Consider alternatively the formula $\exists \pi .\forall \pi ' .\Box (a_\pi \rightarrow b_{\pi '})$. In this case, $\{a\}\emptyset$ dominates $\{a\}\{a\}$ and $\{b\}\emptyset$ dominates $\{b\}\{b\}$.

For our conference management example formula given in Eq. 1, a trace $\{pc\}\emptyset \{v\}$ dominates $\{pc\}\emptyset \emptyset$ and $\emptyset \{s\}\emptyset$ dominates $\emptyset \emptyset \emptyset$, but $\emptyset \{s\}\emptyset$ and $\{pc\}\emptyset \{v\}$ are incomparable with respect to the dominance relation. These are exactly the results that motivated the analysis.

## Evaluation

We report on experimental results of our implementation RVHyper [21], where traces are given sequentially to the monitor, to detect violations after multiple executions of a program. RVHyper is written in C++. It uses *spot* for building the deterministic monitor automata and the *Buddy* BDD library for handling symbolic constraints. We use the $\text {HyperLTL}$ satisfiability solver EAHyper [18, 19] to determine whether the input formula is reflexive, symmetric, or transitive.

In our experiments, we show that (1) the specification analysis is a cheap preprocessing step that reduces the amount of comparisons corresponding to the factor determined in the analysis, (2) the trace analysis has a small overhead during runtime and is effective when applicable, and (3) both optimizations can be used in combination and their impact is orthogonal.

*Specification analysis* We checked variations of observational determinism, quantitative non-interference [22], equality, a Hamming-distance of 2, and our conference management example for symmetry, transitivity, and reflexivity. The results are depicted in Table 1. The specification analysis comes with low costs (every check was done in under a second), but with a high reward in terms of constructed monitor instances. Figure 9 shows the runtime during the monitoring of a Hamming-distance preserving encoder: analyzing the specification beforehand halves the algorithmic workload by omitting symmetric monitor instantiations.

**Table 1:** Specification analysis for universally quantified hyperproperties

		Symm	trans	refl
ObsDet1	$\forall \pi . \forall \pi '.\; \Box (I_{\pi } = I_{\pi '}) \rightarrow \Box (O_{\pi } = O_{\pi '})$	✓	✗	✓
ObsDet2	$\forall \pi . \forall \pi '.\; (I_{\pi } = I_{\pi '}) \rightarrow \Box (O_{\pi } = O_{\pi '})$	✓	✗	✓
ObsDet3	$\forall \pi . \forall \pi '. (O_\pi = O_\pi ') {\mathcal {W}}(I_\pi \ne I_\pi ')$	✓	✗	✓
QuantNoninf	$\forall \pi _0 \ldots \forall \pi _{c}.~\lnot ((\bigwedge _i I_{\pi _i} = I_{\pi _0}) \wedge \bigwedge _{i \ne j} O_{\pi _i} \ne O_{\pi _j})$	✓	✗	✓
EQ	$\forall \pi . \forall \pi ' .\Box (a_\pi \leftrightarrow a_{\pi '})$	✓	✓	✓
Hamming-2	$\forall \pi . \forall \pi '. (\diamondsuit (I_\pi \nleftrightarrow I_{\pi '}) \rightarrow ((O_\pi \leftrightarrow O_{\pi '})$	✓	✗	✓
ConfMan	$$\forall \pi \forall \pi ' .\big ((\lnot pc_\pi \wedge pc_{\pi '}) \rightarrow \Circle \Box (s_\pi \rightarrow \Circle v_{\pi '})\big )$$$$\wedge \big ((pc_{\pi } \wedge pc_{\pi '}) \rightarrow \Circle \Box (v_\pi \leftrightarrow v_\pi ')\big )$$	✗	✗	✗

**Fig. 8:**
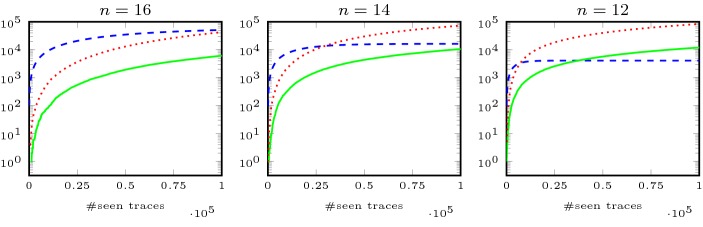
Absolute numbers of violations in red (dotted), number of instances stored in blue (dashed), number of instances pruned in green (solid) for $10^5$ randomly generated traces of length 100, 000. The *y* axis is scaled logarithmically

*Trace analysis* For evaluating our trace analysis, we use a scalable, bounded variation of observational determinism: $\forall \pi .\forall \pi '.\Box _{<n} (I_\pi = I_{\pi '}) \rightarrow \Box _{< n +c} (O_\pi = O_{\pi '})$, where $\Box _{<n} \varphi$ is syntactic sugar that $\varphi$ has to hold until the $(n-1)$-th position of the incoming traces. Figure 8 shows a family of plots for this benchmark class, where *c* is fixed to three. We randomly generated a set of $10^5$ traces. The blue (dashed) line depicts the number of traces that need to be stored, the red (dotted) line the number of traces that violated the property, and the green (solid) line depicts the pruned traces. When *increasing the requirements* on the system, i.e., decreasing *n*, we prune the majority of incoming traces with our trace analysis techniques.

**Fig. 9:**
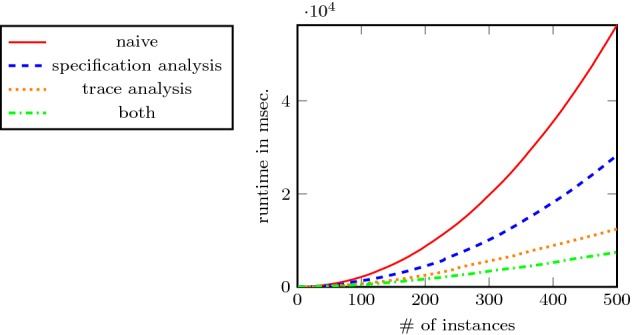
Hamming-distance preserving encoder: runtime comparison of naive monitoring approach with different optimizations and a combination thereof

*Optimizations in combination* We furthermore considered our optimizations in combination. As a first benchmark we monitored whether an encoder preserves a Hamming-distance of 2, which can be encoded as a universally quantified HyperLTL formula [12]: . In Fig. 9 we compare the naive monitoring approach to the monitor using specification analysis and trace analysis, as well as a combination thereof. We randomly built traces of length 50. In each position of the trace, the corresponding bit had a 1% chance to be flipped. Applying our techniques results in a tremendous speed up of the monitoring algorithm.

**Fig. 10:**
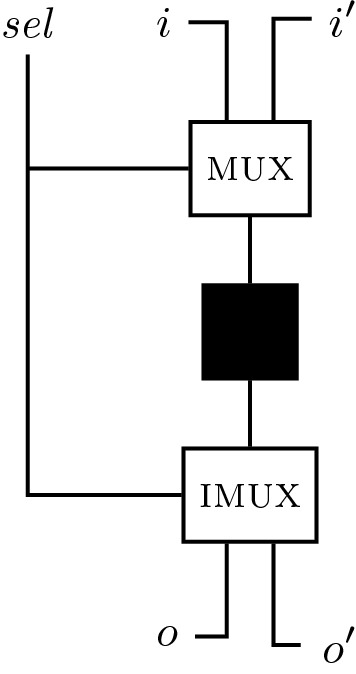
mux circuit with black box

For our second benchmark, we evaluate what effect the similarity of the incoming traces have on our optimizations. For this setup, we considered a black box combinatorial circuit, guarded by a multiplexer that selects between the two input vectors $\mathbf {i}$ and $\mathbf {i}'$ and an inverse multiplexer that forwards the output of the black box either towards $\mathbf {o}$ or $\mathbf {o}'$. The circuit is depicted in Fig. 10. We monitored whether there is a semantic dependency between the in- and outputs, i.e., $\forall \pi _1 \forall \pi _2 .(\mathbf {o}_{\pi _1} = \mathbf {o}_{\pi _2}) {\mathcal {W}}(\overline{\mathbf {i}}_{\pi _1} \ne \overline{\mathbf {i}}_{\pi _2})$, where ${\mathcal {W}}$ is the weak version of the until operator, i.e., $\varphi {\mathcal {W}}\psi :=\Box \varphi \vee \varphi {\mathcal {U}}\psi$. Intuitively, the formula asserts that for every two pairs of execution traces $(\pi _1,\pi _2)$ the value of $\mathbf {o}$ has to be the same until there is a difference between $\pi _1$ and $\pi _2$ in the input vector $\overline{\mathbf {i}}$, i.e., the inputs on which $\mathbf {o}$ may depend. For our experimental evaluation we generated each time 1000 traces of length 30 and varying trace similarity. Specifically the traces of a single configuration were derived by flipping the input bits of a reference input trace with a certain probability after which we simulated the mux circuit on the altered input.

**Fig. 11:**
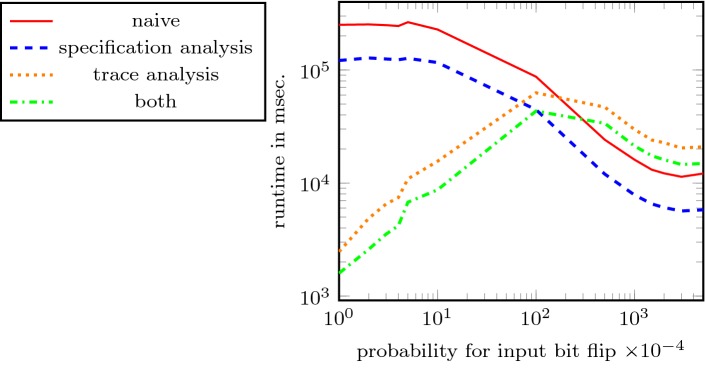
Monitoring of black box circuits: runtime comparison of naive monitoring approach with different optimizations and a combination thereof

Figure 11 shows the total runtime of our monitoring approach with the different optimizations and a combination thereof. Note that both axes are log-scaled. Also note that the x-axis is chosen such that from left to right the trace similarity decreases, that is, there are only few unique traces in the left most configuration up to almost only different traces in the right most one. From the plot we can derive several interesting characteristics of our optimizations that we discuss in the following. As observed in our previous experiments, the specification analysis, if applicable as in this case, is a valuable optimization consistently reducing the runtime and does so also when combined with the trace analysis. As expected the runtime is halved by exploiting symmetry and reflexivity in the formula. From the plot we can also infer that the trace analysis is effective in a context with a majority of redundant traces. For such a highly redundant setup the trace analysis reduces the overall runtime of the monitoring algorithm by several magnitudes. With a decrease of similarity and redundancy in the traces the positive effect of the trace analysis steadily decreases up until the overhead of the trace analysis itself gets noticeable. The decrease in runtime for configurations without trace analysis, which comes with reduced traces similarity, is explained by the fact that the more the input of the monitored traces is different the earlier trace tuples can get pruned as they satisfy the specification and thereby reduce the computational burden of the algorithm. This is also the reason why the configurations with trace analysis show decreasing runtime behavior again as soon as the aforementioned effects dominate the runtime characteristics of the monitoring approach.

## Conclusion

In this article, we considered the problem of monitoring hyperproperties in the temporal logic $\text {HyperLTL}$. We studied the question whether a $\text {HyperLTL}$ formula is monitorable in three different input models, where the traces are either given in parallel or sequentially, and, when given sequentially, may either grow beyond any bound or be limited by a fixed bound. For each input model, we have presented automata-based monitoring algorithms for $\text {HyperLTL}$.

We presented two optimizations tackling different problems in monitoring hyperproperties: The trace analysis minimizes the needed memory, by minimizing the stored set of traces and the specification analysis reduces the algorithmic workload by reducing the number of comparisons between a newly observed trace and the previously stored traces.

We have evaluated our tool implementation RVHyper on several benchmarks. We showed that the optimizations contribute significantly towards the practical monitoring of hyperproperties. Furthermore, we provided a use case on detecting spurious dependencies in hardware designs using RVHyper and compared it against a previous prototype implementation.
